# The nonlinear dynamics and fluctuations of mRNA levels in cell cycle coupled transcription

**DOI:** 10.1371/journal.pcbi.1007017

**Published:** 2019-04-29

**Authors:** Qiwen Sun, Feng Jiao, Genghong Lin, Jianshe Yu, Moxun Tang

**Affiliations:** 1 Center for Applied Mathematics, Guangzhou University, Guangzhou, 510006, China; 2 Department of Mathematics, Michigan State University, East Lansing, Michigan, United States of America; Gladstone Institute (UCSF), UNITED STATES

## Abstract

Gene transcription is a noisy process, and cell division cycle is an important source of gene transcription noise. In this work, we develop a mathematical approach by coupling transcription kinetics with cell division cycles to delineate how they are combined to regulate transcription output and noise. In view of gene dosage, a cell cycle is divided into an early stage $S_{1}$ and a late stage $S_{2}$. The analytical forms for the mean and the noise of mRNA numbers are given in each stage. The analysis based on these formulas predicts precisely the fold change r* of mRNA numbers from $S_{1}$ to $S_{2}$ measured in a mouse embryonic stem cell line. When transcription follows similar kinetics in both stages, r* buffers against DNA dosage variation and r* ∈ (1, 2). Numerical simulations suggest that increasing cell cycle durations up-regulates transcription with less noise, whereas rapid stage transitions induce highly noisy transcription. A minimization of the transcription noise is observed when transcription homeostasis is attained by varying a single kinetic rate. When the transcription level scales with cellular volume, either by reducing the transcription burst frequency or by increasing the burst size in $S_{2}$, the noise shows only a minor variation over a wide range of cell cycle stage durations. The reduction level in the burst frequency is nearly a constant, whereas the increase in the burst size is conceivably sensitive, when responding to a large random variation of the cell cycle durations and the gene duplication time.

## Author summary

Gene transcription in single cells is inherently a stochastic process, resulting in a large variability in the number of transcripts and constituting the phenotypic heterogeneity in cell population. Cell division cycle has global effects on transcriptional outputs, and is thought to be an additional source of transcription noise. In this work, we develop a hybrid model to delineate the combined contribution of transcription activities and cell divisions in the variability of transcript counts. By working with the analytical forms of the mean and the noise of mRNA numbers, we show that if the transcription kinetic rates do not change considerably, then the average mRNA level is increased about 1 to 2 folds from earlier to later cell cycle stages. When transcription homeostasis is attained by varying a single kinetic rate between the two cell cycle stages, we find no significant changes in the transcription noise, and the homeostasis nearly minimizes the noise. In our continuous study on the transcript concentration homeostasis that the transcription level scales with the cellular volume, we find only minor variations of the noise if the homeostasis is maintained either by reducing the transcription burst frequency or by increasing the burst size in late cell cycle phase, in the face of a large cell cycle stage duration variation. The reduction in the burst frequency is relative robust, while the increase in the burst size is conceivably sensitive, to the large random variation of the cell cycle durations and the gene duplication time.

## Introduction

Single cell studies over last decades have shown that gene transcription is inherently a stochastic process in a bursting fashion [1–5]. The transcriptional bursting, whereby a gene promoter transits randomly between short periods of mRNA production and long periods of no productions, has been widely studied and invoked to explain how the fluctuation of mRNA molecules arises among single cells of identical genes [6–8]. Early studies on the origin of variability in gene expression found that the noise is not solely due to the randomness in reactions intrinsic to gene expression [9].

Recent experiments have suggested that cell division cycle is an important source of gene expression noise [10–13]. In virtually all cells, from bacteria to mammalian cells, a conserved class of genes is involved in cell cycle stage-specific gene expression. For instance, *SWI5* and *CLB2* are responsible for mitotic progression, whose transcripts are stable during the interphase, but exhibit a 30-fold increase in degradation in the mitosis phase [12]. In budding yeast, acetylation of histone 3 suppresses transcription activity to buffer changes in DNA dose for expression homeostasis of other genes during DNA replication [13]. During cell division processes, genome duplication involves DNA dosage increase at discrete times in *S* phase, and introduces considerable variations in gene copies [13–15]. Moreover, the time spent between two successive cell-division events [11], the DNA replication catalyzed by DNA polymerases [16, 17], the variation in transcription kinetics between different cell cycle stages [9, 15, 18], and the partition of molecules between two daughter cells [19], are all observed to be stochastic and may contribute to cell-to-cell variability in transcript counts.

It remains largely unexplored how these random events govern mRNA outputs and their fluctuation among individual cells [1]. In this work, we initiate a mathematical approach by coupling the classical two-state model with cell division cycles to delineate the combined contribution of transcription activities and cell divisions in the variability of transcript counts [4, 6, 20]. In view of gene dosage, a cell cycle is divided into $S_{1}$ and $S_{2}$ stages. In each stage, the target gene transits randomly between active and inactive states with constant rates. As usual, we use the mean, the noise, and the noise strength to characterize stochastic gene transcription. For a given random variable *N*, we denote by E[*N*], E[*N*^2^], and Var[*N*] = E[*N* − E[*N*]]^2^ its mean, the second moment, and variance, respectively. Its noise and the noise strength are defined by

$$\begin{array}{c} \eta^{2}\left(N\right)=\frac{\text{Var}[N]}{{\left(\text{E}\left[N\right]\right)}^{2}}=\frac{\text{E}\left[N^{2}\right]-{\left(\text{E}\left[N\right]\right)}^{2}}{{\left(\text{E}\left[N\right]\right)}^{2}},\;\;\text{and}\;\;\Phi \left(N\right)=\frac{\text{Var}[N]}{\text{E}[N]}. \end{array}$$ (1)

We will formulate the master equations for the model and derive the differential equations of the mean and the second moment. The analytical forms of the mean, the noise, and the noise strength at steady-state will be given.

We measure the fold change of mRNA copy numbers from $S_{1}$ to $S_{2}$ by $r^{*}=m_{2}^{*}/m_{1}^{*}$, where $m_{1}^{*}$ and $m_{2}^{*}$ are the mean transcription levels at the two stages. Although r* may take any prescribed value in theory, we find that when the transcription kinetic rates are similar in the two stages, the fold change buffers against the DNA dosage variation and stays within (1, 2), as observed in yeast [12] and mammalian cells [15]. Furthermore, if stage transitions are considerably slower than transcription state transitions and mRNA turnover, then r* ≈ 2. The accuracy of our theoretical results is tested by numerical examples that generate nearly the same fold change measured in a mouse embryonic stem cell line [15]. Increasing either of the cell cycle durations up-regulates transcription with less noise, and rapid transitions between cell cycle stages are a major source of highly noisy transcription. Our numerical examples also demonstrate that transcription homeostasis does not bring significant changes in transcription noise. If transcription homeostasis is attained by varying a single kinetic rate in the two cell cycle stages, then the homeostasis nearly minimizes transcription noise. Motivated by increasing evidences that many cellular processes depend mainly on the concentration rather than the absolute number of enzymes [18, 21, 22], we continue to study the noise profile when the transcript concentration homeostasis is maintained. Our analysis reveals an interesting phenomenon that the transcription noise is relatively stable when the concentration homeostasis is maintained, either by reducing the transcription burst frequency or by increasing the burst size in late cell cycle phase, over a wide range of cell cycle stage durations. The reduction degree in the burst frequency is nearly a constant, while the increase in the burst size is conceivably sensitive, when responding to a large random variation of the cell cycle durations and the gene duplication time.

## Models

### Coupling transcription with cell division cycle

In past two decades, the two-state model has been a prevailing tool to characterize stochastic gene transcription in single cells, from bacteria, yeast, to mammalian cells [4–6, 8, 20, 23]. In the model, as depicted in the diagram

$$\text{gene}\;\text{OFF}\overset{\lambda }{\underset{\gamma }{⇄}}\text{gene}\;\text{ON}\overset{\nu }{\to }\text{mRNA}\overset{\delta }{\to }\emptyset ,$$ (2)

it is postulated that the gene promoter transits randomly between inactive (gene OFF) and active (gene ON) states with constant activation rate λ > 0 and inactivation rate *γ* > 0. The transcripts are produced only when the gene is active with a synthesis rate *ν* > 0, and are turned over with a degradation rate *δ* > 0. Apparently, as the four rates are all assumed to be constants, the transcription described by the model is independent of many important cellular processes such as cell growth and cell division.

**Fig 1:**
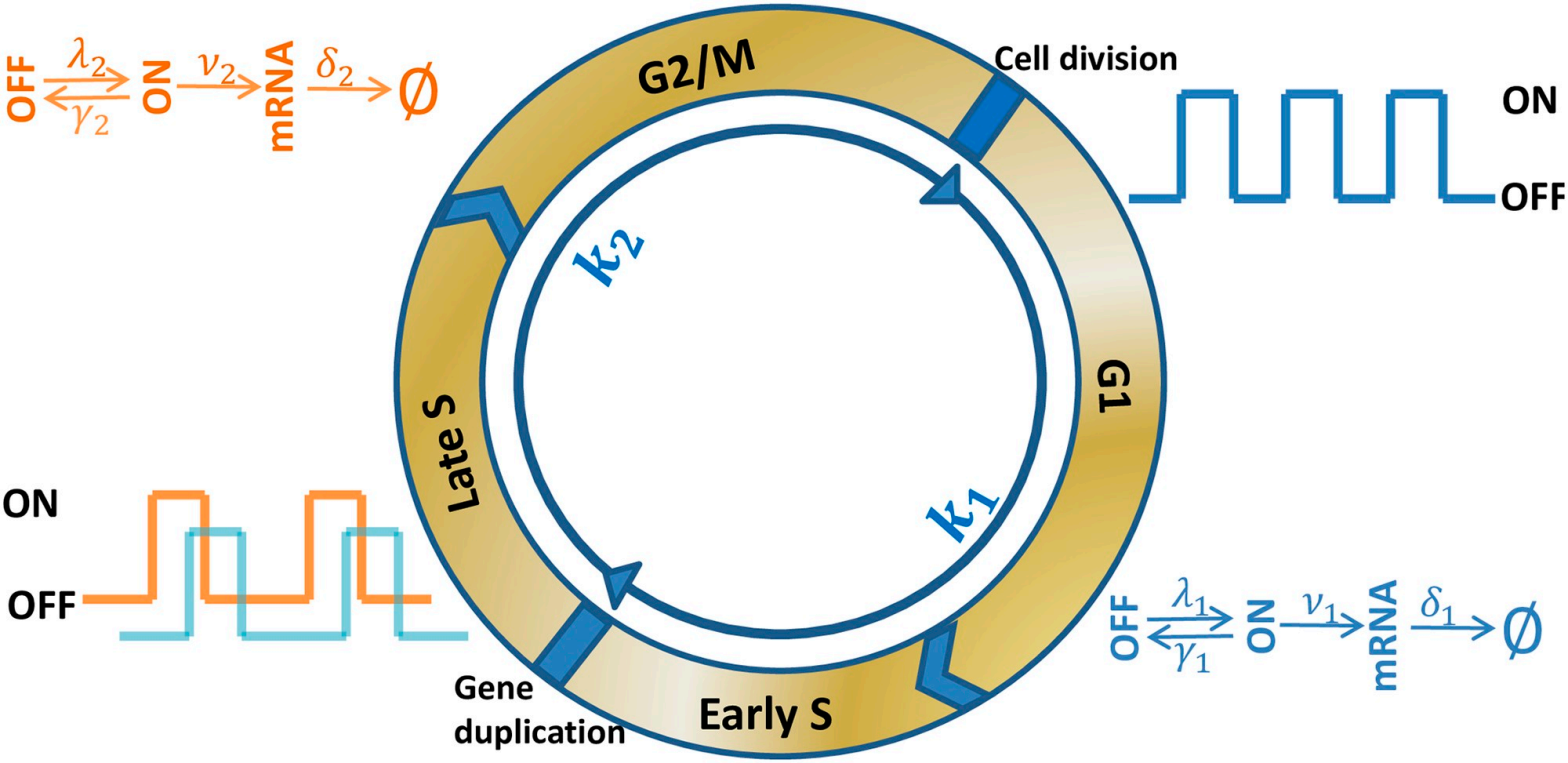
Coupling gene transcription with cell cycle. Actively dividing eukaryote cells go through *G*_1_, *S*, *G*_2_ and *M* phases in one cell cycle. In our model, we divide one cycle into two stages: $S_{1}$ (from last division to gene duplication) and $S_{2}$ (from gene duplication to next division). Cells orderly rotate between $S_{1}$ and $S_{2}$ stages with constant rates *κ*_1_ and *κ*_2_. The promoter transits randomly between active and inactive states, and the transcription kinetics changes with the cell cycle stages. During $S_{1}$ stage, the kinetics is parameterized by activation rate λ_1_, inactivation rate *γ*_1_, synthesis rate *ν*_1_, and mRNA degradation rate *δ*_1_. After DNA replication, the gene is duplicated into two identical copies that are transcribed independently with constant rates λ_2_, *γ*_2_, *ν*_2_ and *δ*_2_ in the same cell during $S_{2}$ stage.

Actively dividing eukaryote cells go through several stages known collectively as the cell division cycle, including Gap 1 phase (*G*_1_) for cell growth, the synthesis phase (*S*) for DNA replication, Gap 2 phase (*G*_2_) for DNA repairing, and the mitotic phase (*M*) for cell division; see Fig 1. During *S* phase, each gene is duplicated into two copies that are transcribed independently in the same cell [15]. During *M* phase, a cell is divided into two daughter cells and residual mRNA molecules are randomly partitioned. Cell division cycle has global effects on mRNA and protein synthesis, and is also an important source of gene expression noise [10–13]. In recent years, many real-time monitoring methods, such as single molecule fluorescent in situ hybridization (smFISH), have been developed to estimate mRNA copy numbers in different cell cycle stages. In mouse embryonic stem cells, nascent Oct4 and Nanog mRNAs were measured in different phases using smFISH method [15]. It was found that the ratio of the average number of mRNA copies in *G*_2_ phase to the average in *G*_1_ phase is 1.28 ± 0.09 for Oct4 mRNA, and 1.51 ± 0.15 for Nanog mRNA. In yeast cells, CLB2 mRNAs accumulate apace in late *S* phase and are degraded almost completely before cytokinesis [12]. From the measurements of [12], we estimated that the median of cytoplasmic CLB2 mRNA copy numbers is ∼10 in *G*_2_/*M* phase, and ∼5 in *S* phase. It remains an essential and widely open question to quantify how the transition of cell cycle phases, the variation of DNA content and transcription kinetics in different phases, and the random partition of mRNAs in daughter cells affect the dynamics and noise of gene transcription.

In this work, we initiate a quantitative approach to this important question by developing a model that couples gene transcription with cell cycles. During DNA replication in *S* phase, the two complementary strands in each double helix are separated and serve as templates for the production of their counterparts. After the completion of the whole DNA replication process, which takes hours in some cells [24], each gene copy is doubled with two copies. Normally, the duplication of a single gene takes much shorter time and is completed within seconds to minutes [17, 25]. For instance, the genome of *Escherichia coli* K12 has ∼ 4.64 million base pairs with ∼ 4375 genes [26], and is replicated at ∼470 ± 180 bp/s [17]. The average duplication time of each gene takes 1.63 ∼ 3.66 seconds. In our model, we treat the short duplication process of our target gene as instantaneous, and accordingly, divide a cell cycle into two stages:

- $S_{1}$ **stage**, consisting of the whole *G*_1_ phase and the early *S* phase until the gene of our interest is duplicated.
- $S_{2}$ **stage**, consisting of the late *S* phase after the gene is duplicated, and *G*_2_/*M* phases.

We make the following assumptions to complete the description of the model:

i The durations *T*_1_ in $S_{1}$ stage and *T*_2_ in $S_{2}$ stage are independently and exponentially distributed with respective rates *κ*_1_ > 0 and *κ*_2_ > 0.
ii At the beginning of each stage, the transcription activity is turned off and the gene remains in the OFF state.
iii In $S_{1}$ stage, the transcription of the single gene copy is described by the two-state model with constant rates λ_1_, *γ*_1_, *ν*_1_ and *δ*_1_.
iv In $S_{2}$ stage, the two identical copies of the gene are transcribed separately and independently, and the transcriptions are described by the two-state model with constant rates λ_2_, *γ*_2_, *ν*_2_ and *δ*_2_.
v At the end of $S_{2}$ stage, each mRNA molecule has an equal probability to be distributed to the two daughter cells; see Fig 1.

We do not assume constant durations in $S_{1}$ and $S_{2}$ stages in (i), because the time spent in each cell cycle phase is often not fixed, and the timing for the duplication of the target gene is random. The cell cycle duration in mouse embryonic stem cells measured by flow cytometry varies in 11 ∼ 16 hours [27, 28], that are roughly distributed in *G*_1_ (26%), *S* (52%), and *G*_2_/*M* (22%) estimated by the percentage of cells in these phases [28]. The times spent in cell cycle phases were also measured by time-lapse microscopy and single cell tracking in T and B lymphocytes from reporter mice, and the total division time data were found to be well approximated by the sum of consecutive independent exponential and Gaussian distributions [11]. We assume that the transcription is turned off at the beginning of each stage, as DNA synthesis is catalyzed by DNA polymerase in nucleosomes, and during late $S_{2}$ stage, the chromatin shrinks into chromosome [29–31]. In either case, transcription factors and RNA polymerase II are usually prevented from reaching to gene promoters to initiate transcription [32]. Assumption (v) is equivalent to the binomial distribution of mRNA molecules in the two daughter cells, which has been assumed in most theoretical models, and supported by recent experiments. The partition in *Escherichia coli* measured by the MS2-GFP reporter strongly supports the assumption that each mRNA copy goes to one of the two daughter cells with equal probability [33].

### The master equations

The transcription state of a gene of our interest in a single cell at a time *t* ≥ 0 can be characterized by the number of active gene copies, the cell cycle stage, and its mRNA copy number. Without loss of generality, we assume that the gene has exactly one copy in $S_{1}$ stage, and two copies in $S_{2}$ stage, in any single cell of an isogenic cell population. We let *I*(*t*) denote the number of active genes in a cell. In $S_{1}$ stage, *I*(*t*) = 0 if the gene is OFF, and *I*(*t*) = 1 if it is ON. In $S_{2}$ stage, *I*(*t*) = 0 if the two gene copies are OFF, *I*(*t*) = 2 if both are ON, and *I*(*t*) = 1 in the remaining cases. We let *U*(*t*) specify the cell cycle stage, with *U*(*t*) = 1 in $S_{1}$ stage, and *U*(*t*) = 2 in $S_{2}$ stage. Let *M*(*t*) denote the mRNA copy number for the gene in one cell. Then the transcription state can be fully quantified by the following joint probabilities

$$\begin{array}{ccc} P_{1}\left(i,m,t\right)=\text{Prob}\{I(t)=i,M(t)=m,U(t)=1\}, & & i=0,1;m=0,1,2,\cdots , \end{array}$$ (3)

$$\begin{array}{ccc} P_{2}\left(i,m,t\right)=\text{Prob}\{I(t)=i,M(t)=m,U(t)=2\}, & & i=0,1,2;m=0,1,2,\cdots . \end{array}$$ (4)

For clarity and simplicity in the following calculations, we assume that all cells in the isogenic population are synchronized at the beginning of $S_{1}$ stage, and count only newly produced mRNA molecules from time zero. Accordingly, we have the initial condition

$$\begin{array}{ccc} & & P_{1}\left(0,0,0\right)=1,\;P_{1}\left(0,m,0\right)=0,\;\;m>0,\\ & & P_{1}\left(1,m,0\right)=P_{2}\left(0,m,0\right)=P_{2}\left(1,m,0\right)=P_{2}\left(2,m,0\right)=0,\;\;m\geq 0. \end{array}$$ (5)

By using the standard procedure in stochastic process, we calculate the time evolutions of these probabilities based on the basic assumptions (i)-(v) in our model and derive the master equations:

P1′(0,m,t)=γ1P1(1,m,t)-(mδ1+λ1+κ1)P1(0,m,t)+(m+1)δ1P1(0,m+1,t)+κ2∑n=m∞(12)n(nm)P2(n,t), (6)

$$\begin{array}{ccc} P_{1}^{\prime }\left(1,m,t\right) & = & \lambda_{1}P_{1}\left(0,m,t\right)-\left(\nu_{1}+m\delta_{1}+\gamma_{1}+\kappa_{1}\right)P_{1}\left(1,m,t\right)\\ & & +\;\nu_{1}P_{1}\left(1,m-1,t\right)+\left(m+1\right)\delta_{1}P_{1}\left(1,m+1,t\right), \end{array}$$ (7)

$$\begin{array}{ccc} P_{2}^{\prime }\left(0,m,t\right) & = & \kappa_{1}P_{1}\left(m,t\right)-\left(m\delta_{2}+2\lambda_{2}+\kappa_{2}\right)P_{2}\left(0,m,t\right)\\ & & +\left(m+1\right)\delta_{2}P_{2}\left(0,m+1,t\right)+\gamma_{2}P_{2}\left(1,m,t\right), \end{array}$$ (8)

$$\begin{array}{ccc} P_{2}^{\prime }\left(1,m,t\right) & = & 2\lambda_{2}P_{2}\left(0,m,t\right)+2\gamma_{2}P_{2}\left(2,m,t\right)+\left(m+1\right)\delta_{2}P_{2}\left(1,m+1,t\right)\\ & & +\;\nu_{2}P_{2}\left(1,m-1,t\right)-\left(\nu_{2}+m\delta_{2}+\lambda_{2}+\gamma_{2}+\kappa_{2}\right)P_{2}\left(1,m,t\right), \end{array}$$ (9)

$$\begin{array}{ccc} P_{2}^{\prime }\left(2,m,t\right) & = & \lambda_{2}P_{2}\left(1,m,t\right)-\left(2\nu_{2}+m\delta_{2}+2\gamma_{2}+\kappa_{2}\right)P_{2}\left(2,m,t\right)\\ & & +\;2\nu_{2}P_{2}\left(2,m-1,t\right)+\left(m+1\right)\delta_{2}P_{2}\left(2,m+1,t\right). \end{array}$$ (10)

The last expression *P*_2_(*n*, *t*) in (6), defined by

$$\begin{array}{c} P_{2}\left(n,t\right)=P_{2}\left(0,n,t\right)+P_{2}\left(1,n,t\right)+P_{2}\left(2,n,t\right), \end{array}$$

gives the probability that the cell resides on $S_{2}$ stage with *n* transcripts, and *P*_1_(*m*, *t*) in (8), defined by *P*_1_(*m*, *t*) = *P*_1_(0, *m*, *t*) + *P*_1_(1, *m*, *t*), represents the probability that the cell resides on $S_{1}$ stage with *m* copies of mRNA molecules. The technical steps leading to (6)–(10) are given in S1 Text.

### The determination of the moment functions

The transcription dynamics of a gene in a cell population is best characterized by the mean value *m*(*t*) = **E**[*M*(*t*)] of the random process *M*(*t*) that counts the number of its mRNA copies. The second moment *μ*(*t*) = **E**[*M*^2^(*t*)] is essential in the calculation of its noise that quantifies the fluctuation of mRNA copy numbers among individual cells. More importantly, as the cell division cycle is integrated into our model, we can extend *m*(*t*) and *μ*(*t*) to the two cell cycle stages $S_{1}$ and $S_{2}$. The comparison of these quantities in the two stages can help us understand how the gene duplication contributes to the variation of transcription levels and noises. To start with, we give the formal definitions of these concepts and present the differential equations that provide a framework from which they can be solved analytically.

For this purpose, we need various probabilities by adding the joint probabilities *P*_*j*_(*i*, *m*, *t*) introduced in (3)–(4) when *i*, *j*, or *m* runs through all possible values. We use a conventional simplification of notations: If any of *i*, *j* and *m* is removed from *P*_*j*_(*i*, *m*, *t*), then the new probability is defined by summing *P*_*j*_(*i*, *m*, *t*) over the range of the removed index. For instance,

$$\begin{array}{c} \begin{array}{c} P_{1}\left(m,t\right)=P_{1}\left(0,m,t\right)+P_{1}\left(1,m,t\right),\\ P_{2}\left(m,t\right)=P_{2}\left(0,m,t\right)+P_{2}\left(1,m,t\right)+P_{2}\left(2,m,t\right) \end{array} \end{array}$$ (11)

are the respective probabilities that the cell resides on $S_{1}$ and $S_{2}$ stages with *m* copies of mRNA molecules, without specifying the promoter state. A further summation of the two probabilities in (11) defines

$$\begin{array}{c} P\left(m,t\right)=P_{1}\left(m,t\right)+P_{2}\left(m,t\right) \end{array}$$ (12)

as the probability that there are *m* copies of mRNA molecules in the cell. Similarly, we can define *P*_1_(*i*, *t*) and *P*_2_(*i*, *t*). To avoid the confusion with these probabilities defined in (11), we change them to *P*_1*i*_(*t*) and *P*_2*i*_(*t*) with

$$\begin{array}{c} P_{1i}\left(t\right)=\sum_{m=0}^{\infty }P_{1}\left(i,m,t\right),\;P_{2i}\left(t\right)=\sum_{m=0}^{\infty }P_{2}\left(i,m,t\right). \end{array}$$ (13)

By adding the probabilities in (13) we have

$$\begin{array}{c} P_{1}\left(t\right)=P_{10}\left(t\right)+P_{11}\left(t\right),\;P_{2}\left(t\right)=P_{20}\left(t\right)+P_{21}\left(t\right)+P_{22}\left(t\right) \end{array}$$ (14)

as the respective probabilities that the cell resides on $S_{1}$ and $S_{2}$ stages. By adding the master Eqs (6)–(10) in *m*, we obtain a closed system of *P*_1*i*_(*t*) and *P*_2*i*_(*t*),

$$\begin{array}{c} \left\{ \begin{array}{cc} P_{10}^{\prime }\left(t\right)= & \kappa_{2}P_{2}\left(t\right)+\gamma_{1}P_{11}\left(t\right)-\left(\lambda_{1}+\kappa_{1}\right)P_{10}\left(t\right),\\ P_{11}^{\prime }\left(t\right)= & \lambda_{1}P_{10}\left(t\right)-\left(\gamma_{1}+\kappa_{1}\right)P_{11}\left(t\right),\\ P_{20}^{\prime }\left(t\right)= & \kappa_{1}P_{1}\left(t\right)+\gamma_{2}P_{21}\left(t\right)-\left(2\lambda_{2}+\kappa_{2}\right)P_{20}\left(t\right),\\ P_{21}^{\prime }\left(t\right)= & 2\lambda_{2}P_{20}\left(t\right)-\left(\lambda_{2}+\gamma_{2}+\kappa_{2}\right)P_{21}\left(t\right)+2\gamma_{2}P_{22}\left(t\right),\\ P_{22}^{\prime }\left(t\right)= & \lambda_{2}P_{21}\left(t\right)-\left(2\gamma_{2}+\kappa_{2}\right)P_{22}\left(t\right). \end{array}\right. \end{array}$$ (15)

The initial condition for this system can be derived by a summation of the initial data given in (5). This linear system of ordinary differential equations with constant coefficients can be solved analytically, and its solution subject to the corresponding initial condition determines uniquely *P*_1*i*_(*t*) and *P*_2*i*_(*t*).

Due to the technical complexity, we break down the process of determining *m*(*t*), *μ*(*t*), and their extensions in $S_{1}$ and $S_{2}$ in several steps, and move most involving calculations to S1 Text.

**Step 1: The determination of the mean level** *m*(*t*): With *P*_1_(*m*, *t*), *P*_2_(*m*, *t*), and *P*(*m*, *t*) defined in (11) and (12), we have

$$\begin{array}{c} m\left(t\right)=E\left[M\left(t\right)\right]=\sum_{m=0}^{\infty }mP\left(m,t\right)=n_{1}\left(t\right)+n_{2}\left(t\right), \end{array}$$ (16)

where

$$\begin{array}{c} n_{1}\left(t\right)=\sum_{k=0}^{\infty }kP_{1}\left(k,t\right),\;\text{and}\;n_{2}\left(t\right)=\sum_{k=0}^{\infty }kP_{2}\left(k,t\right). \end{array}$$ (17)

As we show in S1 Text, *n*_1_(*t*) and *n*_2_(*t*) satisfy the following system of inhomogeneous linear ordinary differential equations with constant coefficients:

$$\begin{array}{c} \left\{ \begin{array}{cc} n_{1}^{\prime }\left(t\right)= & -\left(\delta_{1}+\kappa_{1}\right)n_{1}\left(t\right)+\frac{\kappa_{2}}{2}n_{2}\left(t\right)+\nu_{1}P_{11}\left(t\right),\\ n_{2}^{\prime }\left(t\right)= & \kappa_{1}n_{1}\left(t\right)-\left(\delta_{2}+\kappa_{2}\right)n_{2}\left(t\right)+\nu_{2}[P_{21}\left(t\right)+2P_{22}\left(t\right)]. \end{array}\right. \end{array}$$ (18)

As *P*_11_(*t*), *P*_21_(*t*), and *P*_22_(*t*) can be solved uniquely from (15), we can find *n*_1_(*t*) and *n*_2_(*t*) by solving (18) subject to the initial condition *n*_1_(0) = *n*_2_(0) = 0, and find *m*(*t*) by (16).

**Step 2: The determination of the second moment** *μ*(*t*): Similar to the definition of *m*(*t*) in (16), we have

$$\begin{array}{c} \mu \left(t\right)=E\left[M^{2}\left(t\right)\right]=\sum_{m=0}^{\infty }m^{2}P\left(m,t\right)=\omega_{1}\left(t\right)+\omega_{2}\left(t\right), \end{array}$$ (19)

where

$$\begin{array}{c} \omega_{1}\left(t\right)=\sum_{k=0}^{\infty }k^{2}P_{1}\left(k,t\right),\;\text{and}\;\omega_{2}\left(t\right)=\sum_{k=0}^{\infty }k^{2}P_{2}\left(k,t\right). \end{array}$$ (20)

As we show in S1 Text, the time evolutions of *ω*_1_(*t*) and *ω*_2_(*t*) are given by the system

$$\begin{array}{c} \left\{ \begin{array}{cc} \omega_{1}^{\prime }\left(t\right)= & -(2\delta_{1}+\kappa_{1})\omega_{1}\left(t\right)+\frac{\kappa_{2}}{4}\omega_{2}\left(t\right)+\delta_{1}n_{1}\left(t\right)\\ & +\frac{\kappa_{2}}{4}n_{2}\left(t\right)+\nu_{1}[2n_{11}\left(t\right)+P_{11}\left(t\right)],\\ \omega_{2}^{\prime }\left(t\right)= & \;\kappa_{1}\omega_{1}\left(t\right)-\left(2\delta_{2}+\kappa_{2}\right)\omega_{2}\left(t\right)+\delta_{2}n_{2}\left(t\right)\\ & +\nu_{2}[P_{21}\left(t\right)+2P_{22}\left(t\right)+2n_{21}\left(t\right)+4n_{22}\left(t\right)], \end{array}\right. \end{array}$$ (21)

where

$$\begin{array}{c} n_{1i}\left(t\right)=\sum_{m=0}^{\infty }mP_{1}\left(i,m,t\right),\;\;i=0,1,\;n_{2i}\left(t\right)=\sum_{m=0}^{\infty }mP_{2}\left(i,m,t\right),\;\;i=0,1,2, \end{array}$$ (22)

and

$$\begin{array}{c} n_{1}\left(t\right)=n_{10}\left(t\right)+n_{11}\left(t\right),\;n_{2}\left(t\right)=n_{20}\left(t\right)+n_{21}\left(t\right)+n_{22}\left(t\right). \end{array}$$

Apparently, (21) is not a closed system, and finding *ω*_1_(*t*) and *ω*_2_(*t*) requires the following system of *n*_1*i*_(*t*) and *n*_2*i*_(*t*):

$$\begin{array}{c} \left\{ \begin{array}{cc} n_{10}^{\prime }\left(t\right)= & \frac{\kappa_{2}}{2}n_{2}\left(t\right)-\left(\delta_{1}+\lambda_{1}+\kappa_{1}\right)n_{10}\left(t\right)+\gamma_{1}n_{11}\left(t\right),\\ n_{11}^{\prime }\left(t\right)= & \lambda_{1}n_{10}\left(t\right)+\nu_{1}P_{11}\left(t\right)-\left(\delta_{1}+\gamma_{1}+\kappa_{1}\right)n_{11}\left(t\right),\\ n_{20}^{\prime }\left(t\right)= & \kappa_{1}n_{1}\left(t\right)+\gamma_{2}n_{21}\left(t\right)-\left(\delta_{2}+2\lambda_{2}+\kappa_{2}\right)n_{20}\left(t\right),\\ n_{21}^{\prime }\left(t\right)= & 2\lambda_{2}n_{20}\left(t\right)+2\gamma_{2}n_{22}\left(t\right)+\nu_{2}P_{21}\left(t\right)-\left(\delta_{2}+\lambda_{2}+\gamma_{2}+\kappa_{2}\right)n_{21}\left(t\right),\\ n_{22}^{\prime }\left(t\right)= & \lambda_{2}n_{21}\left(t\right)+2\nu_{2}P_{22}\left(t\right)-\left(\delta_{2}+2\gamma_{2}+\kappa_{2}\right)n_{22}\left(t\right), \end{array}\right. \end{array}$$ (23)

This system is obtained by multiplying (6)–(10) with *m* and then taking sums. As *P*_11_(*t*), *P*_21_(*t*), and *P*_22_(*t*) can be solved from (15), it is a closed system of *n*_1*i*_(*t*) and *n*_2*i*_(*t*). By substituting its unique solution subject to the zero initial condition into (21), we can determine *ω*_1_(*t*) and *ω*_2_(*t*), and therefore the second moment *μ*(*t*).

**Step 3: The moment functions on** $S_{1}$ **and** $S_{2}$ **stages**: To extend the definitions of *m*(*t*) and *μ*(*t*) to the two cell cycle stages $S_{1}$ and $S_{2}$, we define the conditional probabilities

$$\begin{array}{c} p_{1}\left(i,m,t\right)=\text{Prob}\left\{I\left(t\right)=i,M\left(t\right)=m\;|\;U\left(t\right)=1\right\}=\frac{P_{1}\left(i,m,t\right)}{P_{1}\left(t\right)}, \end{array}$$ (24)

$$\begin{array}{c} p_{2}\left(i,m,t\right)=\text{Prob}\left\{I\left(t\right)=i,M\left(t\right)=m\;|\;U\left(t\right)=2\right\}=\frac{P_{2}\left(i,m,t\right)}{P_{2}\left(t\right)}, \end{array}$$ (25)

for the probabilities *P*_1_(*t*) and *P*_2_(*t*) defined in (14). Then

$$\begin{array}{c} p_{1}\left(m,t\right)=p_{1}\left(0,m,t\right)+p_{1}\left(1,m,t\right),\;\;p_{2}\left(m,t\right)=p_{2}\left(0,m,t\right)+p_{2}\left(1,m,t\right)+p_{2}\left(2,m,t\right) \end{array}$$

are the probabilities that there are *m* copies of mRNA molecules when the cell resides on $S_{1}$ or $S_{2}$ stage. The average transcription levels in $S_{1}$ and $S_{2}$ stages are defined by

$$\begin{array}{c} m_{1}\left(t\right)=\sum_{k=0}^{\infty }kp_{1}\left(k,t\right),\;m_{2}\left(t\right)=\sum_{k=0}^{\infty }kp_{2}\left(k,t\right), \end{array}$$ (26)

and the second moments are defined by

$$\begin{array}{c} \mu_{1}\left(t\right)=\sum_{k=0}^{\infty }k^{2}p_{1}\left(k,t\right),\;\mu_{2}\left(t\right)=\sum_{k=0}^{\infty }k^{2}p_{2}\left(k,t\right). \end{array}$$ (27)

By comparing (26) with the definition of *n*_1_(*t*) and *n*_2_(*t*) in (17), and (27) with the definition of *ω*_1_(*t*) and *ω*_2_(*t*) in (20), we find the simple relation

$$\begin{array}{c} m_{1}\left(t\right)=\frac{n_{1}\left(t\right)}{P_{1}\left(t\right)},\;m_{2}\left(t\right)=\frac{n_{2}\left(t\right)}{P_{2}\left(t\right)},\;\mu_{1}\left(t\right)=\frac{\omega_{1}\left(t\right)}{P_{1}\left(t\right)},\;\mu_{2}\left(t\right)=\frac{\omega_{2}\left(t\right)}{P_{2}\left(t\right)}. \end{array}$$ (28)

As a cell is either on $S_{1}$ or on $S_{2}$ stage, we have *P*_1_(*t*) + *P*_2_(*t*) ≡ 1. From the basic assumption (i), the two stages $S_{1}$ and $S_{2}$ transit each other by constant rates *κ*_1_ and *κ*_2_. It implies that *P*_1_(*t*) and *P*_2_(*t*) are simply related by

$$\begin{array}{c} P_{1}^{\prime }\left(t\right)=\kappa_{2}P_{2}\left(t\right)-\kappa_{1}P_{1}\left(t\right)=\kappa_{2}-\left(\kappa_{1}+\kappa_{2}\right)P_{1}\left(t\right). \end{array}$$

This simple equation can also be derived by adding equations in (15). By the assumption that all cells are synchronized on $S_{1}$ initially, we have *P*_1_(0) = 1. Hence

$$\begin{array}{c} P_{1}\left(t\right)=\frac{\kappa_{2}}{\kappa_{1}+\kappa_{2}}+\frac{\kappa_{1}}{\kappa_{1}+\kappa_{2}}\;e^{-(\kappa_{1}+\kappa_{2})t},\;\;\;\;P_{2}\left(t\right)=\frac{\kappa_{1}}{\kappa_{1}+\kappa_{2}}-\frac{\kappa_{1}}{\kappa_{1}+\kappa_{2}}\;e^{-(\kappa_{1}+\kappa_{2})t}. \end{array}$$ (29)

Our methods for finding *n*_1_(*t*) and *n*_2_(*t*) in Step 1, and *ω*_1_(*t*) and *ω*_2_(*t*) in Step 2, combined with (28) and (29), constitute a complete analytical approach for computing the mean values *m*_1_(*t*) and *m*_2_(*t*), and the second moments *μ*_1_(*t*) and *μ*_2_(*t*), in the two cell cycle stages.

## Results

### Analytic expressions of the first and the second moments

Our discussion in the previous section offers a clear analytical approach for finding the mean value *m*(*t*) and the second moment *μ*(*t*) of mRNA number *M*(*t*), along with their extensions to the two cell cycle stages $S_{1}$ and $S_{2}$. However, neither of these functions has a simple analytical expression. For simplicity, we will only present their steady-state values in exact forms, and use their temporal forms in numerical simulations. Although the steady-state values are much simpler than the temporal forms, they are still rather complex and capture the delicate involvement of the system parameters as shown by the next two theorems. For a function *f*(*t*) that has a finite limit as *t* → ∞, we let f* denote its limit.

**Theorem 1** *If the transcription of a gene obeys the model described in* Fig 1, *then the mean transcription level of the gene in a population of isogenic cells at steady-state is*

$$\begin{array}{c} m^{*}=m_{1}^{*}\cdot \frac{\kappa_{2}}{\kappa_{1}+\kappa_{2}}+m_{2}^{*}\cdot \frac{\kappa_{1}}{\kappa_{1}+\kappa_{2}}, \end{array}$$ (30)

*a linear combination of the mean levels* $m_{1}^{*}$ *in* $S_{1}$ *stage and* $m_{2}^{*}$ in $S_{2}$ *stage, and*

$$\begin{array}{c} m_{1}^{*}=\frac{2\nu_{1}\lambda_{1}\left(\delta_{2}+\kappa_{2}\right)\left(\lambda_{2}+\gamma_{2}+\kappa_{2}\right)+2\nu_{2}\lambda_{2}\kappa_{1}\left(\lambda_{1}+\gamma_{1}+\kappa_{1}\right)}{\left[2\left(\delta_{1}+\kappa_{1}\right)\left(\delta_{2}+\kappa_{2}\right)-\kappa_{1}\kappa_{2}\right]\left(\lambda_{1}+\gamma_{1}+\kappa_{1}\right)\left(\lambda_{2}+\gamma_{2}+\kappa_{2}\right)}, \end{array}$$ (31)

$$\begin{array}{c} m_{2}^{*}=\frac{2\nu_{1}\lambda_{1}\kappa_{2}\left(\lambda_{2}+\gamma_{2}+\kappa_{2}\right)+4\nu_{2}\lambda_{2}\left(\delta_{1}+\kappa_{1}\right)\left(\lambda_{1}+\gamma_{1}+\kappa_{1}\right)}{\left[2\left(\delta_{1}+\kappa_{1}\right)\left(\delta_{2}+\kappa_{2}\right)-\kappa_{1}\kappa_{2}\right]\left(\lambda_{1}+\gamma_{1}+\kappa_{1}\right)\left(\lambda_{2}+\gamma_{2}+\kappa_{2}\right)}. \end{array}$$ (32)

**Theorem 2** *If the transcription of a gene obeys the model described in* Fig 1, *then the second moment of its mRNA copy number M*(*t*) *at steady-state is*

$$\begin{array}{c} \mu^{*}=\mu_{1}^{*}\cdot \frac{\kappa_{2}}{\kappa_{1}+\kappa_{2}}+\mu_{2}^{*}\cdot \frac{\kappa_{1}}{\kappa_{1}+\kappa_{2}}, \end{array}$$ (33)

*where* $\mu_{1}^{*}$ *and* $\mu_{2}^{*}$ *are the second moments in* $S_{1}$ *and* $S_{2}$ *stages given by*

$$\begin{array}{c} \mu_{1}^{*}=m_{1}^{*}+\frac{8\nu_{1}\left(\kappa_{2}+2\delta_{2}\right)\cdot m_{s1}^{*}+2\nu_{2}\kappa_{1}\cdot m_{s2}^{*}}{4\left(\kappa_{1}+2\delta_{1}\right)\left(\kappa_{2}+2\delta_{2}\right)-\kappa_{1}\kappa_{2}}, \end{array}$$ (34)

$$\begin{array}{c} \mu_{2}^{*}=m_{2}^{*}+\frac{8\nu_{1}\kappa_{2}\cdot m_{s1}^{*}+8\nu_{2}\left(\kappa_{1}+2\delta_{1}\right)\cdot m_{s2}^{*}}{4\left(\kappa_{1}+2\delta_{1}\right)\left(\kappa_{2}+2\delta_{2}\right)-\kappa_{1}\kappa_{2}}, \end{array}$$ (35)

*with*

$$\begin{array}{cc} m_{s1}^{*}= & \frac{\left(\delta_{1}+\lambda_{1}+\kappa_{1}\right)m_{1}^{*}-\kappa_{1}m_{2}^{*}/2}{\delta_{1}+\lambda_{1}+\gamma_{1}+\kappa_{1}}, \end{array}$$ (36)

$$\begin{array}{cc} m_{s2}^{*}= & \frac{\left(\delta_{2}+\kappa_{2}+2\lambda_{2}\right)m_{2}^{*}-\kappa_{2}m_{1}^{*}+2\nu_{2}p_{22}^{*}}{\delta_{2}+\lambda_{2}+\gamma_{2}+\kappa_{2}}, \end{array}$$ (37)

*and* $p_{22}^{*}=2\lambda_{2}^{2}/[\left(\kappa_{2}+\lambda_{2}+\gamma_{2}\right)\left(\kappa_{2}+2\lambda_{2}+2\gamma_{2}\right)]$.

The proofs of Theorems 1 and 2 are given in S1 Text. By using definition (1), combined with the analytical expressions (31) and (32) of the stationary mean transcription levels, and (34) and (35) for the second moments, we derive the noise strengths of mRNA copy numbers in $S_{1}$ and $S_{2}$ as

$$\begin{array}{c} \Phi_{1}^{*}=1-m_{1}^{*}+\frac{1}{m_{1}^{*}}\cdot \frac{8\nu_{1}\left(\kappa_{2}+2\delta_{2}\right)\cdot m_{s1}^{*}+2\nu_{2}\kappa_{1}\cdot m_{s2}^{*}}{4\left(\kappa_{1}+2\delta_{1}\right)\left(\kappa_{2}+2\delta_{2}\right)-\kappa_{1}\kappa_{2}}, \end{array}$$ (38)

$$\begin{array}{c} \Phi_{2}^{*}=1-m_{2}^{*}+\frac{1}{m_{2}^{*}}\cdot \frac{8\nu_{1}\kappa_{2}\cdot m_{s1}^{*}+8\nu_{2}\left(\kappa_{1}+2\delta_{1}\right)\cdot m_{s2}^{*}}{4\left(\kappa_{1}+2\delta_{1}\right)\left(\kappa_{2}+2\delta_{2}\right)-\kappa_{1}\kappa_{2}}. \end{array}$$ (39)

The noises $\eta_{1}^{2*}$ and $\eta_{2}^{2*}$ are given by

$$\begin{array}{c} \eta_{1}^{2*}=\frac{1}{m_{1}^{*}}-1+\frac{1}{{\left(m_{1}^{*}\right)}^{2}}\cdot \frac{8\nu_{1}\left(\kappa_{2}+2\delta_{2}\right)\cdot m_{s1}^{*}+2\nu_{2}\kappa_{1}\cdot m_{s2}^{*}}{4\left(\kappa_{1}+2\delta_{1}\right)\left(\kappa_{2}+2\delta_{2}\right)-\kappa_{1}\kappa_{2}}, \end{array}$$ (40)

$$\begin{array}{c} \eta_{2}^{2*}=\frac{1}{m_{2}^{*}}-1+\frac{1}{{\left(m_{2}^{*}\right)}^{2}}\cdot \frac{8\nu_{1}\kappa_{2}\cdot m_{s1}^{*}+8\nu_{2}\left(\kappa_{1}+2\delta_{1}\right)\cdot m_{s2}^{*}}{4\left(\kappa_{1}+2\delta_{1}\right)\left(\kappa_{2}+2\delta_{2}\right)-\kappa_{1}\kappa_{2}}. \end{array}$$ (41)

### The fold change of transcripts

#### The broad range of the fold change

We use $r^{*}=m_{2}^{*}/m_{1}^{*}$, the ratio of the mean transcription levels in the two stages, as a measure to quantify the fold change of gene transcription from $S_{1}$ stage to $S_{2}$ stage [12, 15, 34]. Our formulas (31) and (32) imply

$$\begin{array}{c} r^{*}=\frac{m_{2}^{*}}{m_{1}^{*}}=\frac{\nu_{1}\lambda_{1}\kappa_{2}\left(\lambda_{2}+\gamma_{2}+\kappa_{2}\right)+2\nu_{2}\lambda_{2}\left(\delta_{1}+\kappa_{1}\right)\left(\lambda_{1}+\gamma_{1}+\kappa_{1}\right)}{\nu_{1}\lambda_{1}\left(\delta_{2}+\kappa_{2}\right)\left(\lambda_{2}+\gamma_{2}+\kappa_{2}\right)+\nu_{2}\lambda_{2}\kappa_{1}\left(\lambda_{1}+\gamma_{1}+\kappa_{1}\right)}. \end{array}$$ (42)

All ten system parameters are involved in this formula. If the transcripts are turned over extremely fast in $S_{1}$ stage such that *δ*_1_ dominates all other parameter values, then r* can be made arbitrarily large. On the contrary, if the transcripts are turned over extremely fast in $S_{2}$ stage, then r* can be made sufficiently small. Thus r* could take any arbitrary positive number as parameter values vary, and the range of the fold change predicted by (42) is the whole set (0, ∞) of positive numbers.

In many bacterial cells, it has been observed that the transcription kinetic rates of some genes remain the same in the whole cell cycle [35], that is,

$$\begin{array}{c} \nu_{i}=\nu ,\;\delta_{i}=\delta ,\;\lambda_{i}=\lambda ,\;\gamma_{i}=\gamma ,\;\;i=1,2. \end{array}$$ (43)

If (43) holds, then (42) can be simplified to

$$\begin{array}{c} r^{*}=\frac{2\left(\delta +\kappa_{1}\right)\left(\lambda +\gamma +\kappa_{1}\right)+\kappa_{2}\left(\lambda +\gamma +\kappa_{2}\right)}{\kappa_{1}\left(\lambda +\gamma +\kappa_{1}\right)+\left(\delta +\kappa_{2}\right)\left(\lambda +\gamma +\kappa_{2}\right)}. \end{array}$$ (44)

What is the range of r* in this case? During *S*/*G*_2_/*M* phases, a cell contains twice as many copies of each gene as that in *G*_1_ phase. Intuitively, one may envisage that the number of mRNA copies in $S_{2}$ stage doubles the number in $S_{1}$ stage and so r* ≈ 2. Our next theorem shows that r* can deviate from 2 largely, and surprisingly, the constraint (43) does not reduce the range of r*.

**Theorem 3** *For any constant C* > 0, *there exist system parameters under the constraint* (43) to make r* = *C*.

This counter-intuitive result predicts that, even when the transcription kinetic rates do not change in different cell cycle phases, the fold change of transcription levels from $S_{1}$ to $S_{2}$ can be made, in theory, arbitrarily large or small. A complete proof of this result is given in S1 Text. In the proof, we specify the stage transition rates *κ*_1_ and *κ*_2_ in terms of the kinetic rates to make r* = *C* for *C* ≤ 1, *C* ∈ (1, 3/2], *C* ∈ (3/2, 2), and *C* ≥ 2 separately. For instance, when *C* ≤ 1, we take

$$\begin{array}{c} \lambda +\gamma =\kappa_{1},\;\;\kappa_{2}=\frac{4\kappa_{1}}{C},\;\;\delta =\frac{\left(16-12C-2C^{3}\right)\kappa_{1}}{C^{3}}>0. \end{array}$$ (45)

Substituting these parameters into (44) gives

$$\begin{array}{cc} r^{*} & =\frac{2\left[\left(16-12C-2C^{3}\right)\kappa_{1}/C^{3}+\kappa_{1}\right]\left(\kappa_{1}+\kappa_{1}\right)+4\kappa_{1}C\cdot \left(\kappa_{1}+4\kappa_{1}/C\right)}{\kappa_{1}\left(\kappa_{1}+\kappa_{1}\right)+\left[\left(16-12C-2C^{3}\right)\kappa_{1}/C^{3}+4\kappa_{2}/C\right]\left(\kappa_{1}+4\kappa_{1}/C\right)}\\ & =\frac{4\left(16-12C-C^{3}\right)\kappa_{1}^{2}/C^{3}+4\left(C+4\right)\kappa_{1}^{2}/C^{2}}{2\kappa_{1}^{2}+\left(C+4\right)\left(16-12C+4C^{2}-2C^{3}\right)\kappa_{1}^{2}/C^{4}}=C. \end{array}$$

We note that (45) requires *κ*_1_ = λ + *γ* and *κ*_2_ ≥ 4*κ*_1_ = 4(λ + *γ*), which corresponds to faster cell cycle stage transitions comparing to gene promoter transitions, and a much shorter $S_{2}$ stage than $S_{1}$ stage. These conditions may not hold regularly in real cells, and a fold change r* < 1 has been rarely observed under the constraint (43). However, there have been measurements reporting a slightly larger than 1-fold change. In 2016, Skinner et al. [15] quantified mature and nascent mRNA levels of *Oct*4 in individual mouse ES cells, and found that the increase from earlier cell cycle stage to later stage was only about 1.3-fold.

Cell cycle stage transitions are often slower than gene promoter state transitions, implying *κ*_1_ < λ + *γ* and *κ*_2_ < λ + *γ*. If we divide the numerator and denominator in (44) by *δ*(λ + *γ*), then we may change (44) to

$$\begin{array}{c} r^{*}=\frac{2+2\kappa_{1}/\left(\lambda +\gamma \right)+\left(2\kappa_{1}+\kappa_{2}\right)/\delta +\left(2\kappa_{1}^{2}+\kappa_{2}^{2}\right)/\left[\delta \left(\lambda +\gamma \right)\right]}{1+\kappa_{2}/\left(\lambda +\gamma \right)+\left(\kappa_{1}+\kappa_{2}\right)/\delta +\left(\kappa_{1}^{2}+\kappa_{2}^{2}\right)/\left[\delta \left(\lambda +\gamma \right)\right]}. \end{array}$$

It is evident that r* ≈ 2 if *κ*_1_, *κ*_2_ are considerably smaller than *δ* and λ + *γ*. It indicates that when the transcription kinetics are unchanged in the two cell cycle stages, and the stage transition is considerably slower than the transcription state transition and mRNA turnover, the mRNA number in $S_{2}$ stage doubles the number in $S_{1}$ stage at steady-state. This doubling property has been observed in several experimental measurements. In 2004, Vintersten et al. [34] reported a strong expression of a developed RFP variant, DsRed.T3, in mouse ES cells, and found that the nascent mRNA level exhibited a 2-fold increase from $S_{1}$ stage to $S_{2}$ stage. In 2011, Trcek et al. [12] measured the cytoplasmic mRNA level of *CLB2* in yeast and also found an approximate 2-fold increase from late *G*_2_/*M* phases to *S* phase.

Next, we characterize the dependance of r* on the stage transition rates *κ*_1_ and *κ*_2_. To emphasize its dependance on these parameters, we write r* = r*(*κ*_1_, *κ*_2_).

**Theorem 4** *Let* (43) *hold*. *Then we have*

a *When κ*_1_ *increases from* 0 *to* ∞, r* increases from r*(0, *κ*_2_) < 2 *until it peaks uniquely and then decreases to approach* 2 *at* ∞. *In particular*, r* > 2 *if and only if*
  $$\begin{array}{c} \kappa_{1}>\kappa_{2}+\frac{\kappa_{2}\left(\kappa_{2}+\lambda +\gamma \right)}{2\delta }. \end{array}$$ (46)
b *When κ*_2_ *increases from* 0 *to* ∞, r* decreases from r*(*κ*_1_, 0) > 2 *until it bottoms out uniquely and then increases to approach* 1 *at* ∞. *In particular*, r* < 1 *if and only if*
  $$\begin{array}{c} \kappa_{2}>2\kappa_{1}+\lambda +\gamma +\frac{\kappa_{1}\left(\lambda +\gamma +\kappa_{1}\right)}{\delta }. \end{array}$$ (47)
c *When κ*_1_ ≤ *κ*_2_, r* *has an upper bound strictly less than* 2.

Theorem 4 gives a precise description on the nonlinear dependance of the fold change r* on the stage transition rates *κ*_1_ and *κ*_2_. Conditions (46) and (47) give the respective sufficient and necessary conditions for r* > 2 and r* < 1. The complete proof of this theorem is given in S1 Text.

## Discussion

### The fold change r* and its dependance on the stage durations

#### The accuracy of estimating the fold change

We use a set of experimental data to test how accurately our formula (42) may help estimate the fold change r* of mRNA copy numbers from the early cell cycle stage $S_{1}$ to the later stage $S_{2}$. In mouse embryonic stem cells, it was observed that the mean OFF duration in the transcription of *Oct*4 increased from 108 min in $S_{1}$ stage to 173 min in $S_{2}$ stage, and the mean ON duration lasted about 56 min in the two stages [15], suggesting

$$\begin{array}{c} \lambda_{1}=0.5556\;{\text{hr}}^{-1},\;\;\lambda_{2}=0.3468\;{\text{hr}}^{-1},\;\;\gamma_{1}=\gamma_{2}=1.0714\;{\text{hr}}^{-1}. \end{array}$$ (48)

In [15], Skinner found that the average duration of $S_{1}$ stage lasted about ∼560 min by using smFISH. Cartwright et al. [36] measured the cell division time in the same cell line under similar growth conditions, and estimated the average time for one cell cycle at ∼13 hr. These data suggest an average $S_{2}$ stage duration at ∼220 min. As the mean life of newly transcribed *Oct*4 mRNA before being converted to mature RNA was found to be close to 3.5 min, we estimate the other parameters as follows:

$$\begin{array}{c} \delta_{1}=\delta_{2}=17.14\;{\text{hr}}^{-1},\;\;\kappa_{1}=0.1071\;{\text{hr}}^{-1},\;\;\kappa_{2}=0.2727\;{\text{hr}}^{-1}. \end{array}$$ (49)

As the synthesis rate remains about the same in the two cell cycle stages, we can simplify (42) to

$$\begin{array}{c} r^{*}=\frac{\lambda_{1}\kappa_{2}\left(\lambda_{2}+\gamma_{2}+\kappa_{2}\right)+2\lambda_{2}\left(\delta_{1}+\kappa_{1}\right)\left(\lambda_{1}+\gamma_{1}+\kappa_{1}\right)}{\lambda_{1}\left(\delta_{2}+\kappa_{2}\right)\left(\lambda_{2}+\gamma_{2}+\kappa_{2}\right)+\lambda_{2}\kappa_{1}\left(\lambda_{1}+\gamma_{1}+\kappa_{1}\right)}. \end{array}$$ (50)

Substituting (48) and (49) into (50) gives r* = 1.2791, which matches precisely the experimental measurement r* = 1.28 ± 0.09 observed in [15].

#### The condensation of r* within [1, 2]

Although Theorem 1 predicts that r* could take any positive value even if the transcription kinetic rates remain the same in all cell cycle phases, we use numerical simulation to demonstrate that it is more likely to observe r* ∈ [1, 2] in some cells. As suggested by [15] in the transcription of *Oct*4 gene in mouse embryonic stem cells, we fix

$$\begin{array}{cc} & \lambda =0.5556\;\;{\text{hr}}^{-1},\;\gamma =1.0714\;\;{\text{hr}}^{-1},\;\nu =1.89\;\;{\text{min}}^{-1},\;\delta =0.14\;\;{\text{hr}}^{-1}. \end{array}$$ (51)

**Fig 2:**
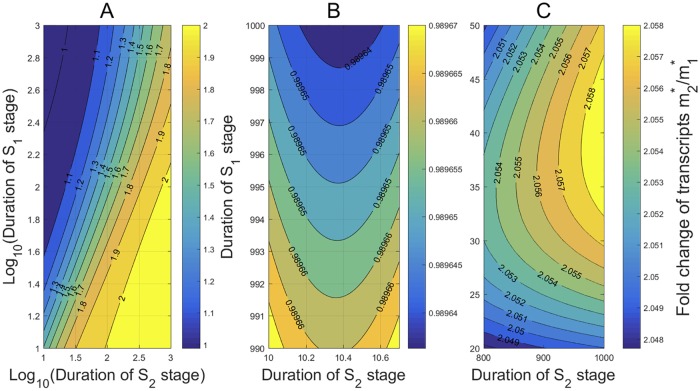
The condensation of r* within [1, 2] and its dependance on the stage durations. All kinetic rates are estimated from [15] in the transcription of *Oct*4 gene in mouse embryonic stem cells. (A) For almost all the $S_{1}$ and $S_{2}$ stage durations ranging from 10 to 1, 000 min, r* is within a narrow range [1, 2]. When 1/*κ*_1_ ≫ 1/*κ*_2_ (or 1/*κ*_1_ ≪ 1/*κ*_2_), r* varies slowly over a narrow region around 1 (or 2). When the two durations are close, r* changes rapidly over (1.1, 2). (B) When the $S_{1}$ stage duration is far greater than the $S_{2}$ stage duration, r* < 1 and is very close to 1. (C) When the $S_{2}$ stage duration is far greater than the $S_{1}$ stage duration, r* > 2 and is very close to 2.

The mean OFF and ON durations are ∼108 min and ∼56 min, respectively, and the mean mRNA lifetime is ∼7.14 hr. To characterize the dependance of the ratio r* on the stage durations, we let the mean $S_{1}$ stage duration 1/*κ*_1_ and the $S_{2}$ stage duration 1/*κ*_2_ vary from 10 to 1, 000 min. The contours of r* on stage durations are shown in Fig 2, where the durations are rescaled in logarithm in Fig 2A. Very interestingly, the contours show that the ratio r* is within the narrow range [1, 2] over almost all the durations, in a good agreement with many experimental measurements [9, 13, 15, 34]. It is also seen that for fixed $S_{2}$ stage duration, r* decreases and tends to a stable value (≈ 1) when the $S_{1}$ stage duration increases. On the other hand, for fixed $S_{1}$ stage duration, r* increases and tends to a larger stable value (≈ 2) when the $S_{2}$ stage duration increases.

To see more clearly how r* changes in the limit cases, we enlarged the left upper corner of Fig 2A in Fig 2B, and the right lower corner of Fig 2A in Fig 2C. The contours in Fig 2B and 2C exhibit some minor deviations from our observation above for Fig 2A. In Fig 2B, where the $S_{1}$ stage duration is far greater than the $S_{2}$ stage duration, r* < 1 and is very close to 1. Also, for fixed large $S_{1}$ duration, r* displays a non-monotone growth as claimed by Theorem 4(b) when the $S_{2}$ duration increases from 10 min to 10.6 min. In Fig 2C, where the $S_{2}$ duration is far greater than the $S_{1}$ duration, r* > 2 and is very close to 2. For fixed large $S_{2}$ duration, r* displays a non-monotone growth as claimed by Theorem 4(c) when the $S_{1}$ duration increases from 20 min to 45 min.

#### The dependance of the fold change on the timing of gene duplication

In the discussion above, the cell cycle duration changes synchronously when the durations of $S_{1}$ or $S_{2}$ change. In some cases, the cell division time may not change significantly, while the variation of $S_{1}$ and $S_{2}$ durations is mainly caused by the random timing of gene duplication that may occur in the early *S* phase or at the end of *S* phase. During *S* phase, DNA synthesis is catalyzed by DNA polymerases [17, 37], and the genome is replicated at a stable rate on leading and lagging strands [25]. In cell cycle-synchronized budding yeast, the total cell division time is ∼70 min, comprising by ∼35 min *G*_1_ phase, ∼18 min *S* phase and ∼17 min *G*_2_/*M* phase [13]. We examine how the mean levels $m_{1}^{*}$, $m_{2}^{*}$ and the ratio r* change when the gene duplication time increases from the start of *S* phase at 35 min to the end of *S* phase at 53 min, and transcripts are produced and turned over with the rates:

$$\begin{array}{c} \nu_{1}=2.52\;{\text{min}}^{-1},\;\nu_{2}=2.14\;{\text{min}}^{-1},\;\delta_{1}=\delta_{2}=1.18\;{\text{hr}}^{-1}. \end{array}$$

We simplify our approach by taking λ_1_, λ_2_ → ∞ and obtain from (31) and (32)

$$\begin{array}{c} m_{1}^{*}=\frac{2\nu_{1}\left(\delta_{2}+\kappa_{2}\right)+2\nu_{2}\kappa_{1}}{2\left(\delta +\kappa_{1}\right)\left(\delta +\kappa_{2}\right)-\kappa_{1}\kappa_{2}},\;m_{2}^{*}=\frac{2\nu_{1}\kappa_{2}+4\nu_{2}\left(\delta +\kappa_{1}\right)}{2\left(\delta +\kappa_{1}\right)\left(\delta +\kappa_{2}\right)-\kappa_{1}\kappa_{2}}. \end{array}$$

It follows that

$$\begin{array}{c} r^{*}=\frac{\nu_{1}\kappa_{2}+2\nu_{2}\left(\delta +\kappa_{1}\right)}{\nu_{1}\left(\delta +\kappa_{2}\right)+\nu_{2}\kappa_{1}}. \end{array}$$

**Fig 3:**
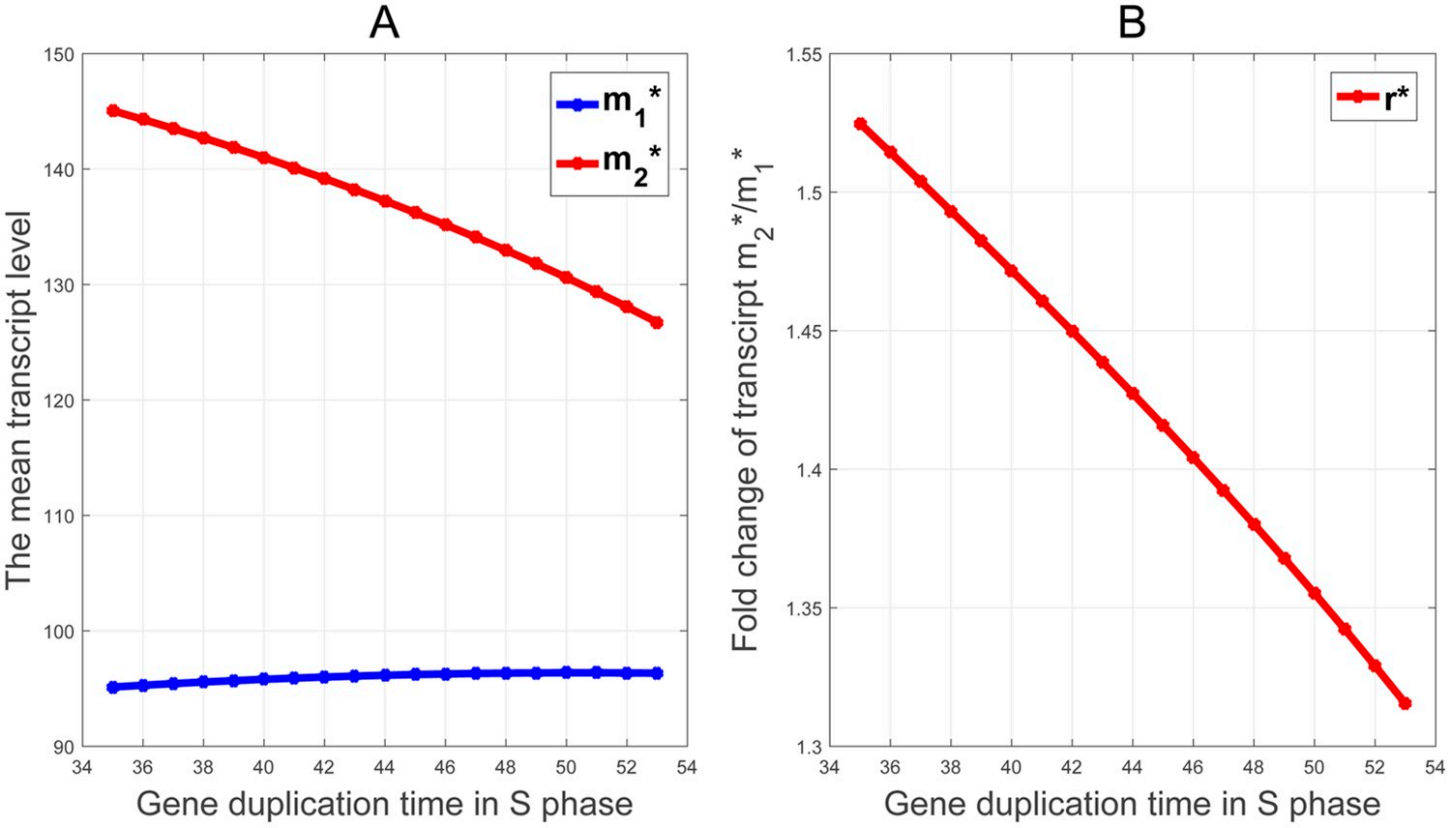
The dependance of the mean mRNA copy numbers $m_{1}^{*}$, $m_{2}^{*}$ and the ratio r* on the timing of gene duplication during *S* phase. (A) The mean level $m_{1}^{*}$ stays nearly at a constant level, while $m_{2}^{*}$ decreases linear as the gene duplication time increases from 35 min to 53 min in *S* phase. (B) The ratio r* decreases almost linearly from 1.53 to 1.31 as the gene duplication time increases.

As shown in Fig 3A, although the mean transcription level $m_{2}^{*}$ in $S_{2}$ decreases in the gene duplication time as we expect, it is surprisingly to see that the mean level $m_{1}^{*}$ in $S_{1}$ remains almost a constant. Very interestingly, as shown in Fig 3B, the ratio r*, which changes moderately in a narrow range from 1.31 to 1.53, decreases almost linearly in the duplication time.

### The effect of cell cycles on transcription noise

Our gene transcription model coupling with cell division cycles offers six quantities to characterize the fluctuations of mRNA numbers in single cells: The noise *η*^2^* and the noise strength Φ* in cells without referring to cell cycle stages, along with $\eta_{1}^{2*}$ and $\Phi_{1}^{*}$ in $S_{1}$ stage, and $\eta_{2}^{2*}$ and $\Phi_{2}^{*}$ in $S_{2}$ stage. Theorems 1 and 2 provide the basic formulas by which these quantities can be computed from the system parameters.

The relations between these quantities are far more complicated than our intuition may envisage. We use simple example to demonstrate the delicacy of their relations: Assume (43) and fix the kinetic rates as in (51), and take the stage transition rates *κ*_1_ = *κ*_2_ = 1/1250 hr^−1^. Then applying Theorem 2, (38) and (39) gives $\Phi_{1}^{*}=43.2008$ and $\Phi_{2}^{*}=43.8410$. Since $\Phi_{1}^{*}$ and $\Phi_{2}^{*}$ are nearly equal, one might expect by intuition that Φ* is about equal to each of $\Phi_{1}^{*}$ and $\Phi_{2}^{*}$, which is in conflict with

$$\begin{array}{c} \Phi^{*}=89.3329>\Phi_{1}^{*}+\Phi_{2}^{*} \end{array}$$

obtained by using Theorems 1 and 2. Moreover Φ* = 89.3329 given here is significantly higher than Φ* ≈ 1 reported in various single cell measurements, including the classical studies by Taniguchi et al. [38] and Yu et al. [39]. In these studies, the genes were active most of time, so that m* and *η*^2^* exhibited a strict reciprocal relation, implying Φ* ≈ 1. The stochastic switching between gene promoter ON and OFF states, combined with transitions between cell cycle stages, may induce much noisier transcriptions.

Due to the wide range of the six noise measures and their complex relations, we will discuss their profiles in three special cases below:

1. The transcription kinetic rates remain constants in all cell cycle phases.
2. The transcription homeostasis is maintained that $m_{1}^{*}=m_{2}^{*}$.
3. The concentration homeostasis is maintained that the mean transcription levels scale with the average volumes in the two stages.

#### The noise profile with constant kinetic rates

DNA replication during cell division cycles induces a doubling of gene copy numbers from $S_{1}$ to $S_{2}$. In bacteria, the transcription kinetic rates of many genes do not change considerably in different cell cycle phases [13]. In this case, mRNA production follows gene dosage with a rapid increase after DNA replication. In a sharp contrast, the response of the transcription noises to the temporal variation of gene dosage is rather complicated and few results have been given to elucidate the complexity. Let the transcription kinetic rates remain constants in all cell cycle phases and fix the rate constants as in (51). We use numerical simulations based on our analytical formulas to depict the profiles of the transcription noise measures and their relations by varying the cell cycle durations over the large time interval (10 min, 10^4^ min).

**Fig 4:**
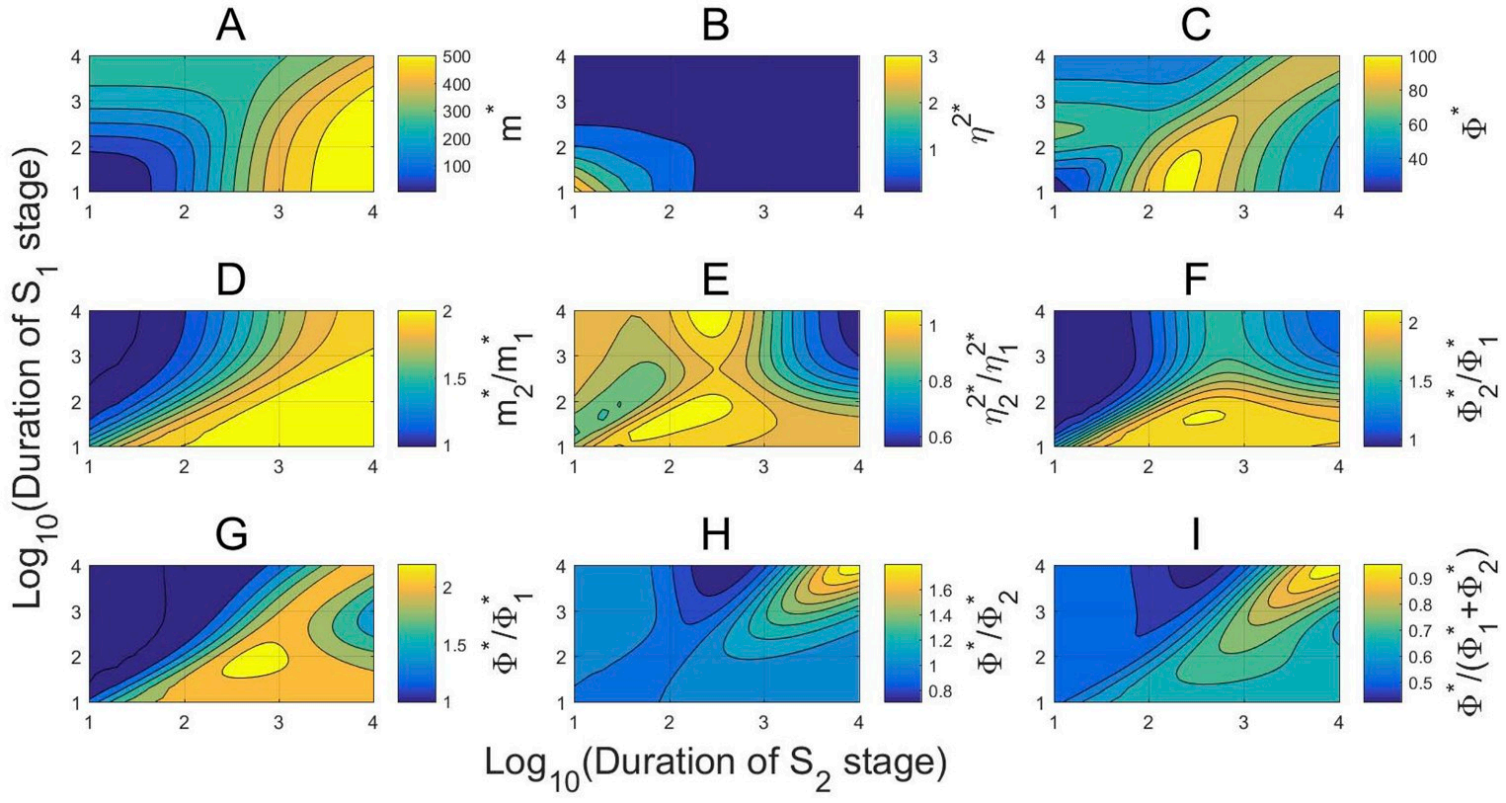
The nonlinear behavior of the transcription noises. The transcription kinetics are specified by (43) and (51), and the stage durations vary from 10 to 10^4^ min. (A) The mean m* increases in the cell cycle durations. (B) The noise *η*^2^* is about equally sensitive to the variation of the stage durations and decreases from 3 to 0.5 as the stage durations increase. (C) The noise strength Φ* varies widely from 20 to 100 with a highly nonlinear dependance on the stage durations. (D)-(F) The ratios $m_{2}^{*}/m_{1}^{*}$, $\Phi_{2}^{*}/\Phi_{1}^{*}\in \left[1,2\right]$, and $\eta_{2}^{2*}/\eta_{1}^{2*}\in \left[0.5,1\right]$ over most durations. (G)-(I) Φ* is larger than both $\Phi_{1}^{*}$ and $\Phi_{2}^{*}$ but less than $\Phi_{1}^{*}+\Phi_{2}^{*}$ over most durations.

In Fig 4A, the contours of the mean transcription level m* in cells display a simple and monotonic variation over the stage durations. It increases in both stage durations and the increase is more sensitive on the second stage duration. Similarly, the contours of the noise *η*^2^* also display a simple and monotonic variation over the stage durations. However, it decreases in both stage durations and the dependance on the two durations are nearly symmetric. Such an inverse relation between m* and *η*^2^* has been observed in many living cell measurements and theoretical studies [23, 35], and a strict reciprocal relation between them has been observed in Taniguchi et al. [38] and Yu et al. [39] with Φ* ≈ 1. Nevertheless, as the transcription specified by (51) is frequently interrupted by long OFF periods, the contours of the noise strength Φ* in Fig 4C indicate that Φ* is significantly higher than 1 with Φ* ∈ [20, 100]. Moreover, Φ* displays a highly nonlinear, non-monotonic variation over the cell cycle stages. Intriguingly, among the durations shown in Fig 4C, Φ* peaks in a small region when $S_{1}$ stage lasts between 10 and 100 minutes on average, and $S_{2}$ stage lasts between 125 and 250 minutes.

In Fig 4D–4F, the fold changes $m_{2}^{*}/m_{1}^{*}$, $\eta_{2}^{2*}/\eta_{1}^{2*}$, and $\Phi_{2}^{*}/\Phi_{1}^{*}$ from $S_{1}$ to $S_{1}$ all vary within a narrow range with $m_{2}^{*}/m_{1}^{*}$, $\Phi_{2}^{*}/\Phi_{1}^{*}\in \left[1,2\right]$, and $\eta_{2}^{2*}/\eta_{1}^{2*}\in \left[0.5,1\right]$. The right upper corners in Fig 4D–4F seem to suggest that, as both stage durations become large,

$$\begin{array}{c} m_{2}^{*}/m_{1}^{*}\approx 2,\;\eta_{2}^{2*}/\eta_{1}^{2*}\approx 1/2,\;\Phi_{2}^{*}/\Phi_{1}^{*}\approx 1. \end{array}$$ (52)

Indeed, (52) can be supported by simple mathematical calculations. By taking limits in (31) and (32), we obtain

$$\begin{array}{c} \lim_{\kappa_{1}\to 0}m_{1}^{*}\left(\kappa_{1},\kappa_{2}\right)=\frac{\nu \lambda }{\delta (\lambda +\gamma )}=\frac{1}{2}\lim_{\kappa_{2}\to 0}m_{2}^{*}\left(\kappa_{1},\kappa_{2}\right). \end{array}$$

and verify the first part of (52). Taking limits in (34) and (35) gives

$$\begin{array}{c} \lim_{\kappa_{1}\to 0}\mu_{1}^{*}\left(\kappa_{1},\kappa_{2}\right)=\frac{\nu \lambda }{\delta (\lambda +\gamma )}[1+\frac{\nu (\delta +\lambda )}{\delta (\delta +\lambda +\gamma )}], \end{array}$$

and

$$\begin{array}{c} \lim_{\kappa_{2}\to 0}\mu_{2}^{*}\left(\kappa_{1},\kappa_{2}\right)=\frac{2\nu \lambda }{\delta (\lambda +\gamma )}[1+\frac{\nu }{\delta }\cdot (\frac{\lambda }{\lambda +\gamma }+\frac{\delta +\lambda }{\delta +\lambda +\gamma })]. \end{array}$$

By the definition of the noise strength in (1), we find

$$\begin{array}{c} \lim_{\kappa_{1}\to 0}\Phi_{1}^{*}\left(\kappa_{1},\kappa_{2}\right)=\lim_{\kappa_{2}\to 0}\Phi_{2}^{*}\left(\kappa_{1},\kappa_{2}\right)=1+\frac{\nu \gamma }{\left(\lambda +\gamma )(\delta +\lambda +\gamma \right)} \end{array}$$ (53)

and verify the third part in (52). Consequently, the second part in (52) is also verified since the noise equals the noise strength divided by the mean.

The approximate identities in (52) indicate that when the cell cycle stage transitions are sufficiently slow, the mean transcription levels are doubled and the noises are halved from $S_{1}$ to $S_{2}$, while the noise strengths remain about the same. However, this simple statement is invalid in general as shown by the contours in Fig 4D–4F, where $\eta_{2}^{2*}/\eta_{1}^{2*}$ and $\Phi_{2}^{*}/\Phi_{1}^{*}$ display highly nonlinear and wild variations.

In Fig 4G–4I, we compare Φ* with $\Phi_{1}^{*}$, $\Phi_{2}^{*}$, and $\Phi_{1}^{*}+\Phi_{2}^{*}$. In most of time,

$$\begin{array}{c} \Phi_{1}^{*}<\Phi^{*}<2\Phi_{1}^{*},\;\Phi_{2}^{*}<\Phi^{*}<2\Phi_{2}^{*},\;\left(\Phi_{1}^{*}+\Phi_{2}^{*}\right)/2<\Phi^{*}<\Phi_{1}^{*}+\Phi_{2}^{*}. \end{array}$$

However, these estimates do not hold universally. For instance, Fig 4H indicates that $\Phi^{*}/\Phi_{2}^{*}$ can be as low as 0.8 and so $\Phi^{*}<\Phi_{2}^{*}$ in some cases. Also, we have shown one example early that $\Phi^{*}>\Phi_{1}^{*}+\Phi_{2}^{*}$ may hold. Overall, Φ* is rarely identical to either $\Phi_{1}^{*}$ or $\Phi_{2}^{*}$, and stays between $\max\{\Phi_{1}^{*},\Phi_{2}^{*}\}$ and $\Phi_{1}^{*}+\Phi_{2}^{*}$ most of time.

#### The noise profile in gene transcription homeostasis

It has been observed repeatedly that gene transcription in eukaryotic cells, ranging from yeast to mammals, has a limited dependency on DNA dosage [13]. Cells have a DNA dosage-compensating mechanism to precisely reduce mRNA production in late cell cycle stage, resulting in a gene transcription homeostasis that overall transcription remains constant across $S_{1}$ and $S_{2}$ stages [18]. Various compensating mechanisms for gene transcription homeostasis have been found [12, 13, 15, 18], including reducing transcription activation or mRNA synthesis rates, and increasing inactivation or mRNA degradation rates in $S_{2}$ stage. For all of the genes measured in foreskin fibroblast cells, Padovan-Merhar et al. [18] found that the number of active sites per gene copy in $S_{2}$ stage was approximately half of that in $S_{1}$. In mouse embryonic stem cells, Skinner et al. [15] found that the activation rates of *Oct4* and *Nanog* genes were reduced from 0.5556 hr^−1^ and 0.1124 hr^−1^ in $S_{1}$ stage to 0.3468 hr^−1^ and 0.08 hr^−1^ in $S_{2}$ stage, respectively. In cell cycle-synchronized budding yeast, Voichek et al. [13] found the reduction of mRNA synthesis rate during *S* phase. In yeasts, Trcek et al. [12] found a 30-fold increase in the mRNA degradation rates of *SWI5* and *CLB2* during prometaphase/metaphase.

Mathematically, we define the gene transcription homeostasis by $m_{1}^{*}=m_{2}^{*}$ that the mean transcription levels in $S_{1}$ and $S_{2}$ stages remain the same. If the homeostasis is brought by varying a single pair of corresponding kinetic rates, then substituting (31) and (52) into $m_{1}^{*}=m_{2}^{*}$ yields the relations of the two varied rates as follows:

$$\begin{array}{ccc} \nu_{2} & = & \nu_{1}\cdot \frac{\delta (\lambda +\gamma +\kappa_{2})}{\left(2\delta +\kappa_{1}\right)\left(\lambda +\gamma +\kappa_{1}\right)}, \end{array}$$ (54)

$$\begin{array}{ccc} \delta_{2} & = & \delta_{1}\cdot \frac{2(\lambda +\gamma +\kappa_{1})}{\lambda +\gamma +\kappa_{2}}+\frac{\kappa_{1}\left(\lambda +\gamma +\kappa_{1}\right)}{\lambda +\gamma +\kappa_{2}}, \end{array}$$ (55)

$$\begin{array}{ccc} \frac{1}{\lambda_{2}} & = & \frac{1}{\lambda_{1}}\cdot \frac{\left(2\delta +\kappa_{1}\right)\left(\gamma +\kappa_{1}\right)}{\delta (\gamma +\kappa_{2})}+\frac{\delta +\kappa_{1}}{\delta (\gamma +\kappa_{2})}. \end{array}$$ (56)

$$\begin{array}{ccc} \gamma_{2} & = & \gamma_{1}\cdot \frac{2\delta +\kappa_{1}}{\delta }+\frac{\delta \lambda +2\delta \kappa_{1}+\lambda \kappa_{1}+\kappa_{1}^{2}-\delta \kappa_{2}}{\delta }, \end{array}$$ (57)

In (54)–(57), the parameters with no subscripts are assumed to be independent of cell cycle stages. It is seen that *ν*_2_ and *ν*_1_ change proportionally, whereas *δ*_2_ and *δ*_1_, *γ*_2_ and *γ*_1_, and the two OFF durations depend linearly.

**Fig 5:**
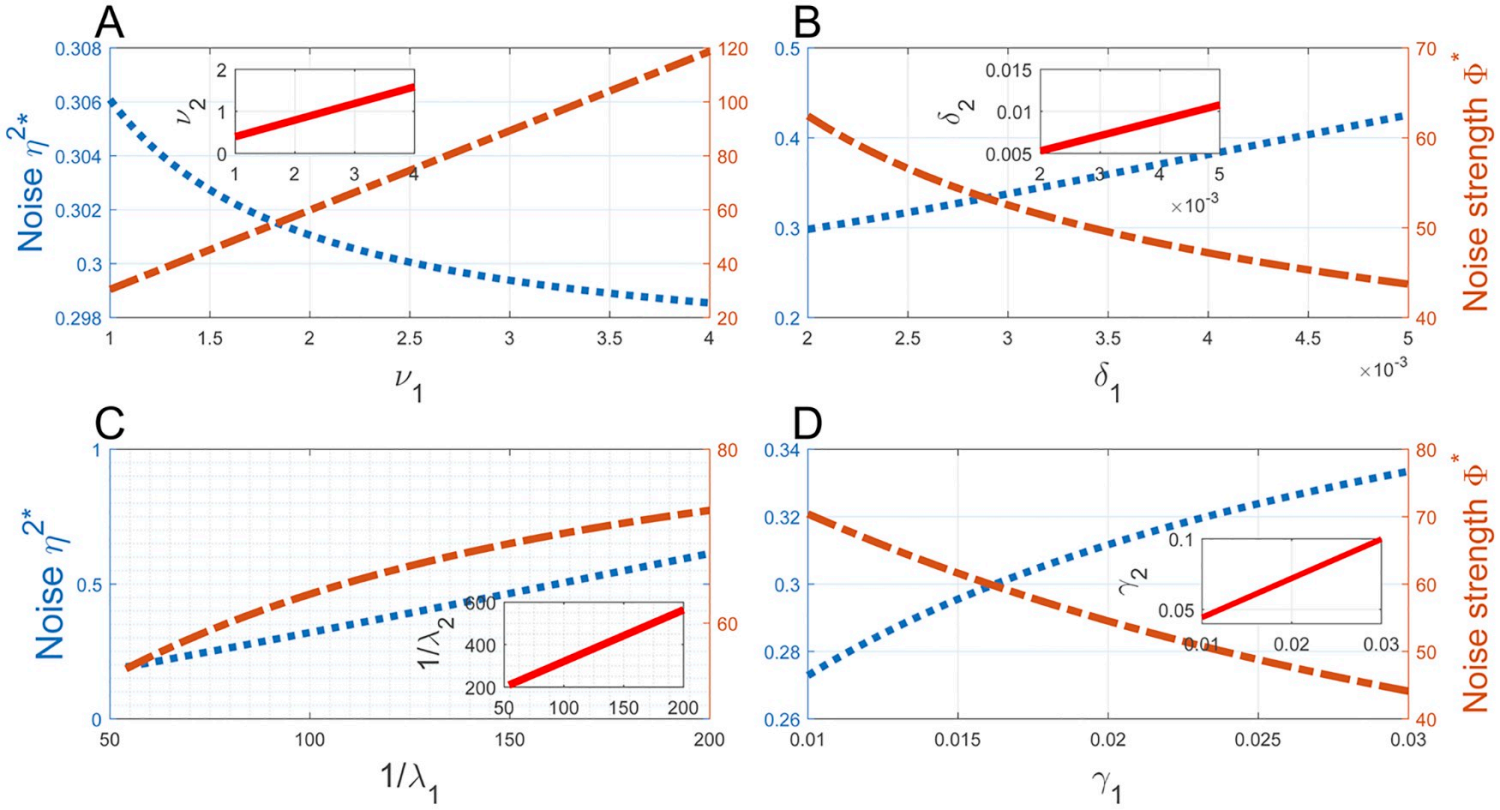
The noise profile in gene transcription homeostasis. The inserts in (A)-(D) depict the linear relations (54)–(57), where *ν*_1_, *δ*_1_, λ_1_, and *γ*_1_ have the same unit min^−1^ and vary near the corresponding values specified in (51). Both *η*^2^* and Φ* change monotonically on these intervals in opposite directions, except in (C) where they both decrease in λ_1_. The noise *η*^2^* takes small values near 0.30 in all panels and has only insignificant variations. The noise strength Φ* takes large values but also shows insignificant variations except in Fig 5A.

We test how the noise *η*^2^* and the noise strength Φ* respond when the parameter pair defined in each of (54)–(57) change linearly to maintain the homeostasis. We again fix the parameters with no subscripts in (54)–(57) as in (51), and let

$$\begin{array}{c} \kappa_{1}^{-1}=560\;\;\text{min},\;\kappa_{2}^{-1}=220\;\;\text{min}, \end{array}$$ (58)

that are approximately the average duration of $S_{1}$ and $S_{2}$ stages in a mouse embryonic stem cell line [15, 36]. In Fig 5A–5D, we vary *ν*_1_, *δ*_1_, λ_1_, and *γ*_1_ near the corresponding parameter values specified in (51), with *ν*_1_ ∈ [1, 4] in (A), *δ*_1_ ∈ [2 × 10^−3^, 5 × 10^−3^] in (B), λ_1_ ∈ [0.005, 0.02] in (C), and *γ*_1_ ∈ [0.01, 0.03] in (D), all sharing the same unit min^−1^. The linear relations of *ν*_1_ and *ν*_2_, *δ*_1_ and *δ*_2_, 1/λ_1_ and 1/λ_2_, and *γ*_1_ and *γ*_2_ defined by (54)–(57) are depicted in the inserts of these panels. It is easily seen that both *η*^2^* and Φ* vary monotonically on these intervals. Interestingly, *η*^2^* and Φ* change in opposite directions in Fig 5A, 5B and 5D, and they both decrease in the activation rate λ_1_ in Fig 5C. The noise *η*^2^* takes small values near 0.30 in all panels and shows only insignificant variations. The noise strength Φ* takes considerably larger values but the variation is also insignificant, except in Fig 5A where it increases from about 30 to 120 when *ν*_1_ increases from 1 to 4. Overall, these data suggest that if the transcription homeostasis is induced by varying a single pair of corresponding kinetic rates, then the variation of the rates does not bring significant changes in transcription noise.

**Fig 6:**
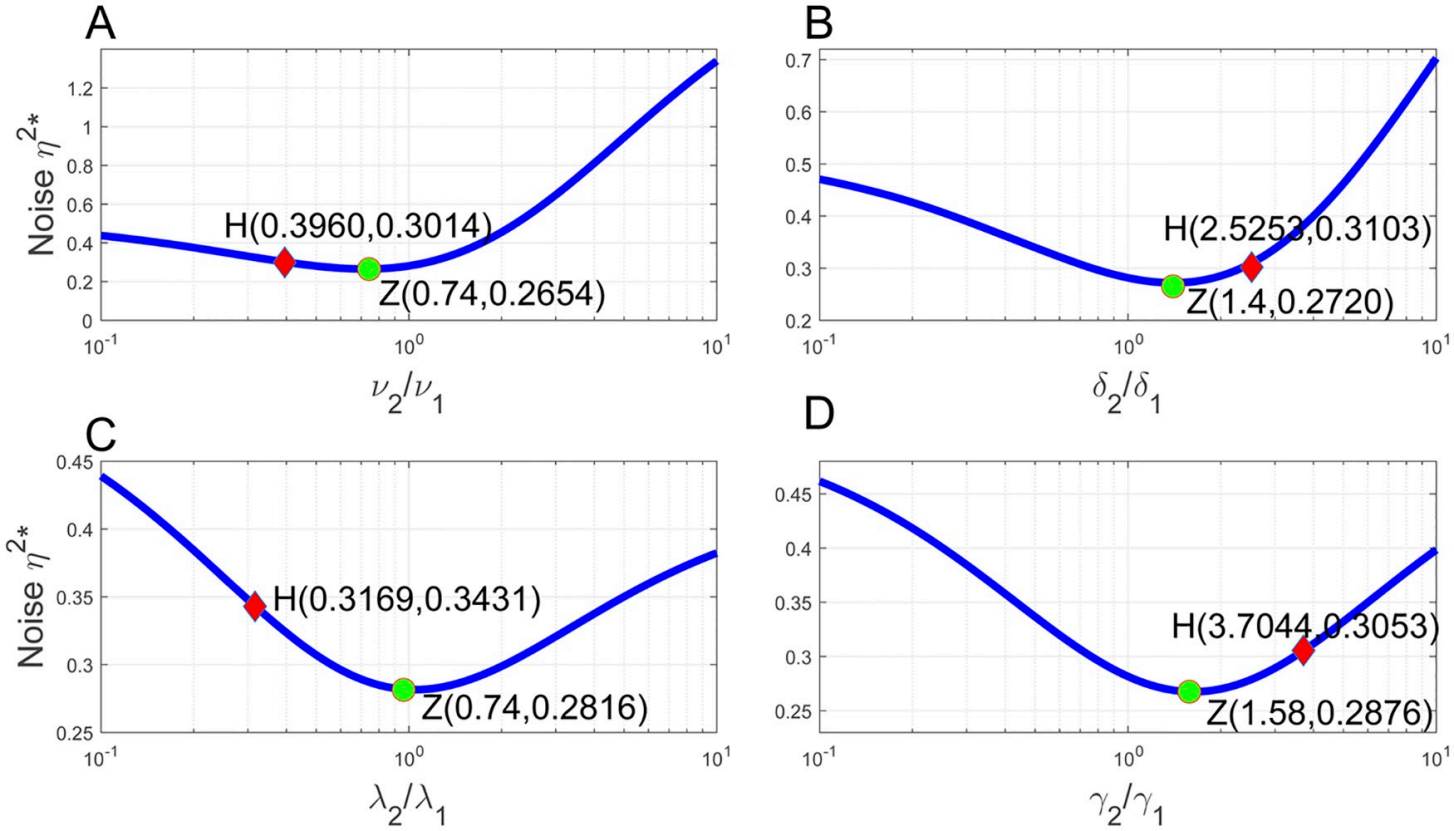
Transcription homeostasis nearly minimizes transcription noise. The ratios *ν*_2_/*ν*_1_, *δ*_2_/*δ*_1_, λ_2_/λ_1_, and *γ*_2_/*γ*_1_ increase from 0.1 to 10 in (A)-(D), whereas other parameters are kept as in (51) and (58). In (A)-(D), the point H at which transcription homeostasis is reached and the noise minimizing point Z stay very close.

We examine the relation between the transcription noise *η*^2^* and transcription homeostasis further by varying *ν*_2_/*ν*_1_, *δ*_2_/*δ*_1_, λ_2_/λ_1_, and *γ*_2_/*γ*_1_ from 0.1 to 10 in Fig 6A–6D. All other parameters are kept as in (51) and (58). We label the points at which transcription homeostasis is reached as H, and the noise minimizing points as Z. In all panels from (A)-(D), it is seen that the two points stay very close. It suggests that if transcription homeostasis is attained by varying a single kinetic rate in the two cell cycle stages, then the homeostasis nearly minimizes transcription noise.

#### The noise profile in transcript concentration homeostasis

Cells reproduce by cell division. During cell mitosis, newly divided daughter cells are naturally smaller than their parent cells. Thus, a cell in $S_{2}$ stage is normally larger than itself in $S_{1}$ stage, although it has also been reported that cellular volumes are weakly related to cell cycle phases [18]. If the transcription homeostasis is maintained as we discussed above, then the transcript concentration in a cell decreases as it grows from $S_{1}$ to $S_{2}$ stage. However, in agree with the paradigm that biochemical reaction rates are determined mostly by the concentration of reactants and enzymes, increasing evidences have shown that many cellular processes depend on the concentration of gene expression products rather than their absolute numbers [18, 21, 22]. In cell populations with a large variability in cellular volumes, the numbers of most molecules need to scale with volumes to maintain a stable concentration for proper cellular functions [18, 21].

Let *V*_1_ and *V*_2_ denote the average volumes of individual cells on $S_{1}$ and $S_{2}$ stages, respectively, and let

$$\begin{array}{c} r_{v}=V_{2}/V_{1} \end{array}$$

denote the fold change of the volumes on the two stages. Recall that $r^{*}=m_{2}^{*}/m_{1}^{*}$ is the fold change of the mean transcription levels at steady-state. We define the transcript concentration homeostasis by

$$\begin{array}{c} r^{*}=r_{v}^{*}, \end{array}$$ (59)

that is, the mean transcription levels scale with the volumes on both cell cycle stages.

We need to find an analytical formula of *r*_*v*_ in terms of the cell cycle stage transition rates *κ*_1_ and *κ*_2_. By combining it with the expression of r* in (42) and the identity (59), we obtain an explicit relation of the system parameters for the transcript concentration homeostasis. The expression of *r*_*v*_ depends on how cells grow and how the two cell cycle stage durations distribute. Various cell growth models have been proposed [22, 40–42], including the prevailing exponential growth model that cell volumes grow exponentially. Assume that a newly divided cell has a volume *V*_0_ = 1 at time *t* = 0. Then the cellular volume *V*(*t*) during the first cell cycle is given by

$$\begin{array}{c} V\left(t\right)=e^{at}, \end{array}$$ (60)

for a constant growth rate *a* > 0. Let (0, *T*_1_) be the duration of $S_{1}$, and (*T*_1_, *T*_1_ + *T*_2_) be the duration of $S_{2}$. We assume that the cell volume is doubled at the end of the cell cycle. Then *V*(*T*_1_ + *T*_2_) = 2. Recall that *T*_1_ and *T*_2_ are independently and exponentially distributed with rates *κ*_1_ and *κ*_2_. With these specifications, we have

$$\begin{array}{c} a=\frac{\kappa_{1}+\kappa_{2}-\sqrt{\kappa_{1}^{2}+\kappa_{2}^{2}}}{2},\;r_{v}=\frac{\kappa_{2}}{\kappa_{2}-a}=\frac{2\kappa_{2}}{\sqrt{\kappa_{1}^{2}+\kappa_{2}^{2}}-\kappa_{1}+\kappa_{2}}. \end{array}$$ (61)

We note that *a* increases in both *κ*_1_ and *κ*_2_, and *r*_*v*_ ∈ (1, 2). When *κ*_1_ → 0, the cell stays sufficiently long in $S_{1}$ and has little growth in $S_{2}$. In this case, (61) gives *r*_*v*_ → 1 as we expect. On the other hand, *r*_*v*_ → 2 as *κ*_2_ → 0.

To prove (61), we begin with the observation that

$$\begin{array}{c} r_{v}=\frac{\text{E}[V\left(T_{1}+T_{2}\right)]}{\text{E}[V\left(T_{1}\right)]}, \end{array}$$

where E denotes expectation. We omit the detail of deriving this identity, which follows from the independent exponential distributions of *T*_1_ and *T*_2_, the renewal theory in stochastic processes, and Proposition 3.4.5 in [43]. From (60), we obtain

$$\begin{array}{c} \text{E}\left[V\left(T_{1}\right)\right]=\int_{0}^{\infty }V\left(t\right)\kappa_{1}e^{-\kappa_{1}t}dt=\int_{0}^{\infty }\kappa_{1}e^{(a-\kappa_{1})t}dt=\frac{\kappa_{1}}{\kappa_{1}-a}. \end{array}$$

The distribution function of *T*_1_ + *T*_2_ is given by

$$\begin{array}{ccc} F(t) & = & \text{Prob}\{T_{1}+T_{2}\leq t\}\\ & = & \int_{0}^{t}\text{Prob}\left\{T_{1}\leq t-x\right\}d\text{Prob}\left\{T_{2}\leq x\right\}\\ & = & \int_{0}^{t}(1-e^{-\kappa_{1}\left(t-x\right)})\kappa_{2}e^{-\kappa_{2}x}dx\\ & = & 1-\frac{\kappa_{2}}{\kappa_{2}-\kappa_{1}}e^{-\kappa_{1}t}-\frac{\kappa_{1}}{\kappa_{1}-\kappa_{2}}e^{-\kappa_{2}t}, \end{array}$$

when *κ*_1_ ≠ *κ*_2_ [5, 8]. By taking limits, it applies to the case *κ*_1_ = *κ*_2_ as well. Thus

$$\begin{array}{c} E\left[V\left(T_{1}+T_{2}\right)\right]=\int_{0}^{\infty }V\left(t\right)dF\left(t\right)=\frac{\kappa_{1}\kappa_{2}}{\left(\kappa_{1}-a\right)\left(\kappa_{2}-a\right)}. \end{array}$$

Since it also holds that E[*V*(*T*_1_ + *T*_2_)] = 2, we derive

$$\begin{array}{c} \frac{\kappa_{1}\kappa_{2}}{\left(\kappa_{1}-a\right)\left(\kappa_{2}-a\right)}=2 \end{array}$$

from which the expression of *a* in (61) is obtained. The second part in (61) can be verified by dividing the expressions of E[*V*(*T*_1_ + *T*_2_)] and E[*V*(*T*_1_)] directly.

**Fig 7:**
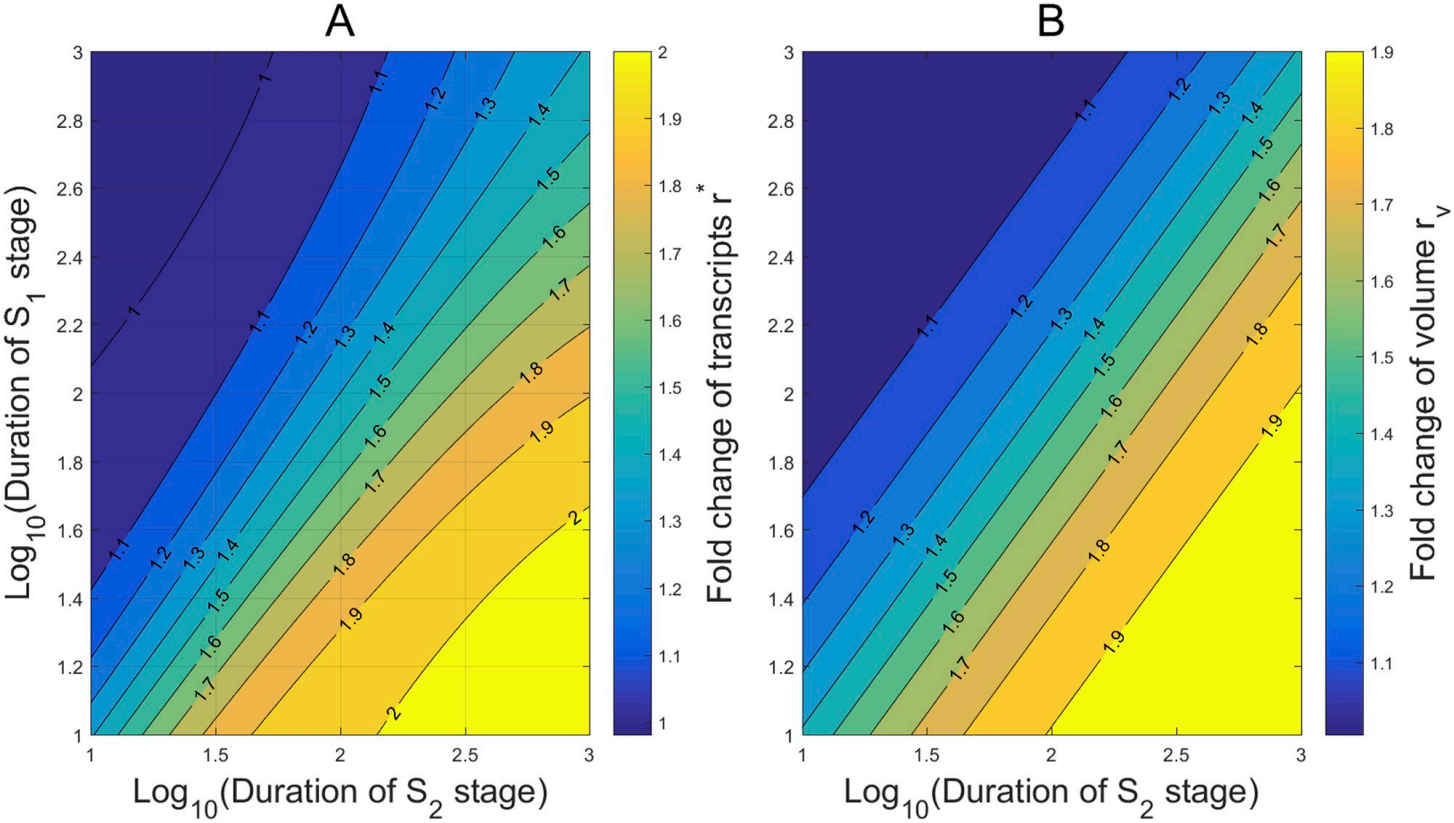
The transcript concentration homeostasis by reducing the transcription burst frequency in *G*_2_ phase. The burst frequency λ_2_ is reduced from 0.5556 in (51) to 0.3445, and other kinetic rates are taken from (51). (A) The contours of the fold change r* for the mean transcription levels from $S_{1}$ to $S_{2}$. (B) The contours of the fold change *r*_*v*_ for the volumes from $S_{1}$ to $S_{2}$. The two sets of contours in (A) and (B) display a large similarity, indicating that the transcript concentration homeostasis is almost reached by reducing the frequency uniformly over most area of the stage durations.

We apply our mathematical formulas (42) and (61) to examine how the transcript concentration homeostasis is attained by varying the transcription frequency λ_*i*_, or the burst size *ν*_*i*_/*γ*_*i*_, *i* = 1, 2, in different cell cycle phases, and how the noise responds to the variation. In a recent interesting study elucidating the mechanism underlying the concentration homeostasis, Padovan-Merhar et al. [18] found a DNA-linked *cis*-acting factor in mammalian cells, that can lead to the reduction of transcription frequency of each gene copy in *G*_2_ phase, and therefore an overall balanced burst frequency in all cell cycle stages. Motivated by this observation, we reduce λ_2_ in (51) from 0.5556 to 0.3445, which is approximately a 38% reduction, and keep other kinetic rates as in (51). As the stage durations vary from 10 min to 1, 000 min, the contours of the fold change r* for the mean transcription levels and the fold change *r*_*v*_ for the volumes are shown in Fig 7. Very interestingly, the contour of r* in Fig 7A and the contour of *r*_*v*_ in Fig 7B are very similar over most area of the stage durations. This surprising similarity suggests that the concentration homeostasis could be reached by merely reducing the transcription burst frequency in *G*_2_ phase, in a uniform reduction degree that is robust against a large variation of cell stage durations and the randomness of gene duplication time.

**Fig 8:**
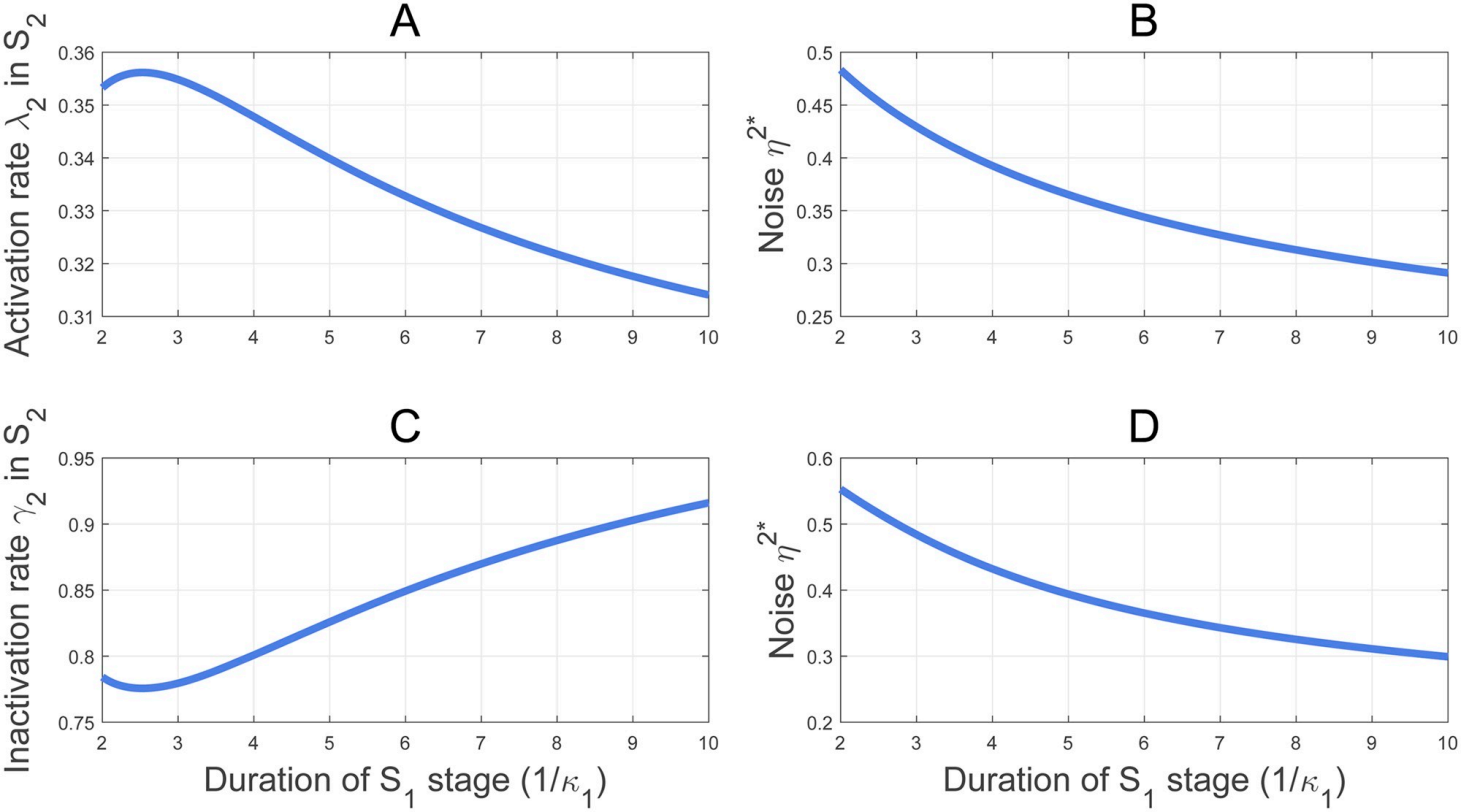
Minor variations of λ_2_, *γ*_2_ and *η*^2^* versus the large variation of the cell cycle durations in the concentration homeostasis. (A) The transcription frequency for the concentration homeostasis stays within (0.315, 0.355) over a large span of the $S_{1}$ duration from 2 to 10 hours; $\kappa_{2}^{-1}=220\;\text{min}$, and other kinetic rates are taken from (58). (B) The noise corresponding to (A) remains within (0.28, 0.48). (C) The inactivation rate *γ*_2_ for the concentration homeostasis with the parameters in (A) except λ_2_ = λ_1_/2 to equalize the burst frequency in $S_{1}$ and $S_{2}$. (D) The noise corresponding to (C) remains within (0.29, 0.56).

To test further the robustness in the reduction of the transcription burst frequency in $S_{2}$ against the variation of cell cycle stage durations, we fix the kinetic rates except λ_2_ as in (51), the duration of $S_{2}$ at $\kappa_{2}^{-1}=220\;\text{min}$ as in (58), but let the duration of $S_{1}$ vary from 2 to 10 hours. By applying (42) and (61) to r* = *r*_*v*_, we determine a unique λ_2_ corresponding to each $S_{1}$ duration for the transcript concentration homeostasis. The curve of λ_2_ versus the $S_{1}$ duration 1/*κ*_1_ is shown in Fig 8A. It is interesting to see that λ_2_ exhibits only a minor variation within a narrow interval (0.315, 0.355) over a large span of the $S_{1}$ duration from 2 to 10 hours. As λ_1_ = 0.5556, this corresponds to a stable reduction, between 36% and 42%, of the transcription frequency from $S_{1}$ to $S_{2}$. We note also that λ_2_ = 0.3445 fixed in Fig 7 is within (0.315, 0.355). More interestingly, the transcription noise sketched in Fig 8B, corresponding to the λ_2_ values of Fig 8A, also exhibits a minor variation within the narrow range (0.28, 0.48).

In Fig 8A and 8B, the transcription burst size *ν*_*i*_/*γ*_*i*_ does not change in $S_{1}$ and $S_{2}$ stages. However, Caveney et al. [21] and Padovan-Merhar et al. [18] found that the transcription burst size increases in larger cells, while the burst frequency in $S_{2}$ shows no dramatic changes. These findings suggest that the increase in the burst size could be responsible for the concentration homeostasis. We are motivated to test how the burst size increase induces the concentration homeostasis by reducing the gene OFF rate *γ*_2_. We set λ_1_, *γ*_1_, and the mRNA synthesis and degradation rates as in (51), and define λ_2_ = λ_1_/2 to equalize the burst frequency on the two stages. As in Fig 8A and 8B, we fix $\kappa_{2}^{-1}=220\;\text{min}$, and let the average duration of $S_{1}$ vary from 2 to 10 hours. For each $S_{1}$ duration 1/*κ*_1_, there is a unique *γ*_2_ determined by the concentration homeostasis identity r* = *r*_*v*_ as shown in Fig 8C. Again, *γ*_2_ displays only a minor variation within the narrow interval (0.78, 0.92) in Fig 8C where the $S_{1}$ duration increases from 2 to 10 hours. However, in contrast to the minor variation of *γ*_2_, the corresponding increase in the bust size is noticeable. As *γ*_1_ = 1.0714, the increase in the burst size in $S_{2}$, given by (*γ*_1_ − *γ*_2_)/*γ*_2_ ranges from 16.45% to 37.35%. Very interestingly, the corresponding transcription noise sketched in Fig 8D does not show a noticeable variation but stays within the narrow range (0.29, 0.56).

The minor variation of *η*^2^* in Fig 8B and 8D suggests that the transcription noise is relatively stable when the transcript concentration homeostasis is maintained, either by reducing the transcription burst frequency or by increasing the burst size in late cell cycle phase, in the face of a large cell cycle stage duration variation. By comparing the profiles of *η*^2^* in Fig 8B and 8D it is also seen that the burst size variation creates a slightly larger noise than reducing the burst frequency, similar to the observation by Caveney et al. [21] in cell-free synthetic reaction chambers. Furthermore, our simulations in Figs 7 and 8A and 8C show that the reduction degree in the burst frequency for the transcript concentration homeostasis is relative robust, while the increase in the burst size is conceivably sensitive, to the random variation of the cell cycle durations.

### Conclusion

Gene transcription involves inherently various probabilistic steps that create fluctuations in mRNA and protein counts [1–3]. The random transitions between the active and inactive promoter states have been widely invoked to explain the fluctuation of mRNA numbers among individual cells of identical genes [6, 7]. Recent experimental studies have revealed that the cell division cycle has global effects on transcriptional outputs, and is thought to be an additional source of transcription noise [10–13].

In this work, we integrated cell division cycles into an extended two-state model to delineate the combined contribution of transcription activities and cell divisions in the variability of transcript counts [4, 6]. In the model, a cell division cycle is divided into $S_{1}$ stage before the duplication of a target gene and the late stage $S_{2}$, on which the durations are independently and exponentially distributed with rates *κ*_1_ and *κ*_2_. When a cell divides, each mRNA molecule has an even chance of being partitioned to one of the two daughter cells. We defined two joint probabilities to quantify the system state, and derived the master equations of their time evolutions. From the master equations we obtained the differential equations of the mean and the second moment of mRNA numbers in single cells. By solving these equations we presented in Theorem 1 the steady-state mean transcription level m* in cells, together with the means $m_{1}^{*}$ and $m_{2}^{*}$ on the two stages. The analytical forms of the second moments are presented in Theorem 2, which in turn help us determine six noise measures: the noise *η*^2^* and the noise strength Φ* without referring to cell cycle stages, along with $\eta_{1}^{2*}$ and $\Phi_{1}^{*}$ in $S_{1}$, and $\eta_{2}^{2*}$ and $\Phi_{2}^{*}$ in $S_{2}$. The fold change of mRNA counts from $S_{1}$ to $S_{2}$ is quantified by $r^{*}=m_{2}^{*}/m_{1}^{*}$. As a cell contains twice as many copies of each gene in $S_{2}$ as that in $S_{1}$, one may envisage by intuition that r* ≈ 2. However, our Theorem 3 shows that r* can take any prescribed value in theory, although we also found that r* ≈ 2 when the transcription kinetics are unchanged in the two stages, and stage transitions are considerably slower than mRNA turnover and transcription state transitions. The dependance of r* on *κ*_1_ and *κ*_2_ is examined deeply in Theorem 4, where the necessary and sufficient conditions for r* < 1 or r* > 2 are identified. In particular, it is proved that r* has an upper bound strictly less than 2 when *κ*_1_ ≤ *κ*_2_.

We tested the accuracy of our analytical results against various experimental data. For a gene in a mouse embryonic stem cell line, our result predicts r* = 1.2791, which offers a good match with r* = 1.28 ± 0.09 measured in [15]. The analysis also indicates that if the transcription kinetics do not change considerably in the two cell cycle stages, then the average mRNA counts increase about 1 to 2 folds from $S_{1}$ stage to $S_{2}$ stage as observed in mouse embryonic cells [15] and yeast [12]. The mean m* increases while the noise *η*^2^* decreases in each of the cell cycle durations. Rapid transitions between cell cycle stages were identified as a major source of highly noisy transcription. Eukaryotic cells have a DNA dosage-compensating mechanism to reduce mRNA production in late cell cycle stage, resulting in gene transcription homeostasis that overall transcription remains constant across $S_{1}$ and $S_{2}$ stages [13, 18]. Our analysis reveals that transcription homeostasis does not bring significant changes in transcription noise. If transcription homeostasis is attained by varying a single kinetic rate in the two cell cycle stages, then the homeostasis nearly minimizes transcription noise. As many cellular processes depend on the concentration of enzymes rather than their absolute numbers for proper cellular function [18, 21, 22], we also studied the noise profile when the transcript concentration homeostasis is maintained that the mean transcription level scales with the cellular volume in $S_{1}$ and $S_{2}$. We found that the transcription noise is relatively stable when the transcript concentration homeostasis is maintained, either by reducing the transcription burst frequency or by increasing the burst size in late cell cycle phase, in the face of a large cell cycle stage duration variation. The reduction degree in the burst frequency is relative robust, while the increase in the burst size is conceivably sensitive, to the large random variation of the cell cycle durations and the gene duplication time.

This work provides one of the first theoretical explorations on how the coupling of stochastic promoter state transitions and cell cycle progressions regulates transcription abundance and noise. It presents a core model for further inclusion of more complex transcription kinetics and cell cycle progressions. The kinetic rates may display large variations in different cell cycle phases or within the same phase, or oscillate periodically in the cell cycle progression [44]. With the expansion of the model, motivated and tested by more upcoming experimental data, the approach initiated here is expected to be developed further to help understand the role played by the cell cycle dependent gene expression in cell functions and cell fate decision [45, 46].

## Supporting information

S1 Text The supporting information consists of two parts: The derivation of differential equations and the proof of theorems. The detailed derivation of master equations and differential equations is given in the former part. Theorems 1-4 are restated in the second part, and the detailed mathematical proofs are given behind these theorems.
(PDF)
