# Supplementary material for: The nonlinear dynamics and fluctuations of mRNA levels in cell cycle coupled transcription
Source: PLoS Comput Biol. 2019 Apr 29;15(4):e1007017. doi: 10.1371/journal.pcbi.1007017 (PMC6508750; doi:10.1371/journal.pcbi.1007017)
Supplement: S1 Text — The detailed derivation of master equations and differential equations is given in the former part. Theorems 1-4 are restated in the second part, and the detailed mathematical proofs are given behind these theorems. (PDF) [file pcbi.1007017.s001.pdf]

SUPPORTING INFORMATION
“The nonlinear dynamics and fluctuations of mRNA levels in cell cycle coupled transcription”

Qiwen Sun[a,b], Feng Jiao[a,b], Genghong Lin[a,b], Jianshe Yu[a] and Moxun Tang[b]

[a] Center for Applied Mathematics, Guangzhou University, Guangzhou, 510006, China

[b] Department of Mathematics, Michigan State University, East Lansing, Michigan, 48824, USA

1 The derivation of differential equations

1.1 The derivation of the master equations

We present the technical steps leading to the master equations

$$
\begin{aligned}
P_1'(0,m,t) &= \gamma_1 P_1(1,m,t) - (m\delta_1 + \lambda_1 + \kappa_1)P_1(0,m,t) \\
&\quad +(m+1)\delta_1 P_1(0,m+1,t) + \kappa_2 \sum_{n=m}^{\infty} \left(\frac{1}{2}\right)^n \binom{n}{m} P_2(n,t), && (1)\\
P_1'(1,m,t) &= \lambda_1 P_1(0,m,t) - (\nu_1 + m\delta_1 + \gamma_1 + \kappa_1)P_1(1,m,t) \\
&\quad +\nu_1 P_1(1,m-1,t) + (m+1)\delta_1 P_1(1,m+1,t), && (2)\\
P_2'(0,m,t) &= \kappa_1 P_1(m,t) - (m\delta_2 + 2\lambda_2 + \kappa_2)P_2(0,m,t) \\
&\quad +(m+1)\delta_2 P_2(0,m+1,t) + \gamma_2 P_2(1,m,t), && (3)\\
P_2'(1,m,t) &= 2\lambda_2 P_2(0,m,t) + 2\gamma_2 P_2(2,m,t) + (m+1)\delta_2 P_2(1,m+1,t) \\
&\quad +\nu_2 P_2(1,m-1,t) - (\nu_2 + m\delta_2 + \lambda_2 + \gamma_2 + \kappa_2)P_2(1,m,t), && (4)\\
P_2'(2,m,t) &= \lambda_2 P_2(1,m,t) - (2\nu_2 + m\delta_2 + 2\gamma_2 + \kappa_2)P_2(2,m,t) \\
&\quad +2\nu_2 P_2(2,m-1,t) + (m+1)\delta_2 P_2(2,m+1,t). && (5)
\end{aligned}
$$

In these equations, the time evolutions of the joint probabilities

$$
\begin{aligned}
P_1(i,m,t) &= \text{Prob}\{I(t)=i, M(t)=m, U(t)=1\}, \quad i=0,1;\ m=0,1,2,\cdots, && (6)\\
P_2(i,m,t) &= \text{Prob}\{I(t)=i, M(t)=m, U(t)=2\}, \quad i=0,1,2;\ m=0,1,2,\cdots, && (7)
\end{aligned}
$$

are expressed by linear combinations of related probabilities. We recall that I, M, and U specify the promoter state, the mRNA copy number of the gene, and the cell cycle stage of a single cell in an isogenic cell population, respectively. Suppose that the gene is OFF and the cell resides on $\mathbb{S}_1$ stage with m copies of mRNA molecules at time $t+h$ for an infinitesimal time increment $h > 0$. Then the basic assumptions (i)-(v) imply that, by discarding the

	Initial State (t)	Terminal State ($t+h$)	Event Probability
(a)	$(\mathrm{OFF}, \mathbb{S}_1, m)$	$(\mathrm{OFF}, \mathbb{S}_1, m)$	$P_1(0,m,t)\cdot(1-\lambda_1 h)(1-\kappa_1 h)(1-m\delta_1 h)$
(b)	$(\mathrm{ON},\ \mathbb{S}_1, m)$	$(\mathrm{OFF}, \mathbb{S}_1, m)$	$P_1(1,m,t)\cdot\gamma_1 h$
(c)	$(\mathrm{OFF}, \mathbb{S}_1, m+1)$	$(\mathrm{OFF}, \mathbb{S}_1, m)$	$P_1(0,m+1,t)\cdot(m+1)\delta_1 h$
(d)	$(*, \mathbb{S}_2, n)$	$(\mathrm{OFF}, \mathbb{S}_1, m)$	$P_2(n,t)\cdot 2^{-n}\binom{n}{m}\cdot\kappa_2 h$

Table 1: **The initial states and transition probabilities toward the terminal state** $(\mathrm{OFF}, \mathbb{S}_1, m)$**.** If the gene is OFF, the cell is in $\mathbb{S}_1$ stage with m copies of the mRNA molecules at $t+h$, then four initial states at time t, listed in (a), (b), (c), (d), can reach the terminal state with a transition probability of order 0 or 1 of the infinitesimal time increment h. In (d), the cell is divided within time interval $(t, t+h)$, and m transcripts are partitioned to one daughter cell from the n transcripts in the mother cell with a probability $2^{-n}\binom{n}{m}$. $P_2(n,t) = P_2(0,n,t) + P_2(1,n,t) + P_2(2,n,t)$ is the probability that the cell resides on $\mathbb{S}_2$ stage with n transcripts.

events with transition probabilities of second or higher order of h, one of the state transition events in Table 1 must occur during the time interval $(t, t+h)$.

Adding the four probabilities listed in Table 1 gives

$$\begin{aligned} P_1(0,m,t+h) =& P_1(0,m,t)(1-\lambda_1 h)(1-\kappa_1 h)(1-m\delta_1 h) + P_1(1,m,t)\gamma_1 h \\ &+ P_1(0,m+1,t)(m+1)\delta_1 h + \sum_{n=m}^{\infty} P_2(n,t)\left(\frac{1}{2}\right)^n \binom{n}{m}\kappa_2 h, \end{aligned}$$

which can be re-organized as

$$\begin{aligned} \frac{P_1(0,m,t+h) - P_1(0,m,t)}{h} =& -(m\delta_1 + \lambda_1 + \kappa_1)P_1(0,m,t) + o(h) + \gamma_1 P_1(1,m,t) \\ &+ (m+1)\delta_1 P_1(0,m+1,t) + \kappa_2 \sum_{n=m}^{\infty}\left(\frac{1}{2}\right)^n \binom{n}{m} P_2(n,t), \end{aligned}$$

where $o(h) \to 0$ as $h \to 0$. By letting $h \to 0$, we obtain

$$\begin{aligned} P_1'(0,m,t) =& \gamma_1 P_1(1,m,t) - (m\delta_1 + \lambda_1 + \kappa_1)P_1(0,m,t) \\ &+ (m+1)\delta_1 P_1(0,m+1,t) + \kappa_2 \sum_{n=m}^{\infty}\left(\frac{1}{2}\right)^n \binom{n}{m} P_2(n,t), \end{aligned}$$

which verifies (6) in the main context. The remaining equations can be verified by the same procedure whose details are omitted for simplicity.

1.2 The derivation of the differential equations of $n_1(t)$ and $n_2(t)$

As shown in the main context, the mean transcription level $m(t)$ in cells has a decomposition $m(t) = n_1(t) + n_2(t)$ with

$$n_1(t) = \sum_{k=0}^{\infty} k P_1(k,t), \quad \text{and} \quad n_2(t) = \sum_{k=0}^{\infty} k P_2(k,t), \tag{8}$$

We present here the process leading to the system of differential equations

$$\begin{cases} n_1'(t) = -(\delta_1+\kappa_1)n_1(t) + \dfrac{\kappa_2}{2}n_2(t) + \nu_1 P_{11}(t), \\ n_2'(t) = \kappa_1 n_1(t) - (\delta_2+\kappa_2)n_2(t) + \nu_2\Big[P_{21}(t) + 2P_{22}(t)\Big]. \end{cases} \tag{9}$$

By using the definition

$$P_1(m,t) = P_1(0,m,t) + P_1(1,m,t), \quad P_2(m,t) = P_2(0,m,t) + P_2(1,m,t) + P_2(2,m,t)$$

we can express $P_1(k,t)$ and $P_2(k,t)$ in (8) as the sums of the basic probabilities defined in (6)-(7). By differentiating $n_1(t)$ in (8), and then substituting (1)-(2), we have

$$n_1'(t) = \sum_{m=0}^{\infty} m\Bigg[\underbrace{\nu_1 P_1(1,m-1,t) - \nu_1 P_1(1,m,t)}_{\text{First term}} + \underbrace{(m+1)\delta_1 P_1(m+1,t) - m\delta_1 P_1(m,t)}_{\text{Second term}}$$
$$- \underbrace{\kappa_1 P_1(m,t)}_{\text{Third term}} + \underbrace{\kappa_2 \sum_{n=m}^{\infty}\left(\frac{1}{2}\right)^n \binom{n}{m} P_2(n,t)}_{\text{Forth term}}\Bigg].$$

By using the definitions of $P_{11}(t)$, $n_1(t)$ and $n_2(t)$, we can simplify the sums of these terms multiplying m as follows. For the first, we have

$$\nu_1 \sum_{m=0}^{\infty} m\Big[P_1(1,m-1,t) - P_1(1,m,t)\Big] = \nu_1 \sum_{m=0}^{\infty} P_1(1,m,t) = \nu_1 P_{11}(t).$$

The second sum is

$$\delta_1 \sum_{m=0}^{\infty}\Big[m(m+1)P_1(m+1,t) - m^2 P_1(m,t)\Big] = -\delta_1 \sum_{m=0}^{\infty} mP_1(m,t) = -\delta_1 n_1(t),$$

and the third sum is simply $\kappa_1 n_1(t)$. Finally, for the last one,

$$\begin{aligned} \kappa_2 \sum_{m=0}^{\infty} m \sum_{n=m}^{\infty}\left(\frac{1}{2}\right)^n \binom{n}{m} P_2(n,t) =& \kappa_2 \sum_{n=0}^{\infty}\left(\frac{1}{2}\right)^n P_2(n,t) \sum_{m=0}^{n} m\binom{n}{m} \\ =& \kappa_2 \sum_{n=0}^{\infty}\left(\frac{1}{2}\right)^n P_2(n,t)\cdot\frac{n\cdot 2^n}{2} = \frac{\kappa_2}{2}\sum_{n=0}^{\infty} nP_2(n,t) = \frac{\kappa_2}{2}n_2(t). \end{aligned}$$

We the help of these simplification, we verify the first equation of (9). The second equation of (9) can be obtained by a similar calculation.

1.3 The derivation of the differential equations of $\omega_1(t)$ and $\omega_2(t)$

The second moment $\mu(t) = \mathbf{E}[M^2(t)]$ of the mRNA copy number $M(t)$ has a decomposition $\mu(t) = \omega_1(t) + \omega_2(t)$ with

$$\omega_1(t) = \sum_{k=0}^{\infty} k^2 P_1(k,t), \quad \text{and} \quad \omega_2(t) = \sum_{k=0}^{\infty} k^2 P_2(k,t). \tag{10}$$

Here we give a brief discussion on the process of deriving the system

$$
\begin{cases}
\omega_1'(t) = -\big(2\delta_1+\kappa_1\big)\omega_1(t) + \dfrac{\kappa_2}{4}\omega_2(t) \\
\qquad\qquad + \delta_1 n_1(t) + \dfrac{\kappa_2}{4} n_2(t) + \nu_1\Big[2n_{11}(t) + P_{11}(t)\Big], \\
\omega_2'(t) = \kappa_1\omega_1(t) - (2\delta_2+\kappa_2)\omega_2(t) + \delta_2 n_2(t) \\
\qquad\qquad + \nu_2\Big[P_{21}(t) + 2P_{22}(t) + 2n_{21}(t) + 4n_{22}(t)\Big],
\end{cases}
\tag{11}
$$

where

$$
n_{1i}(t) = \sum_{m=0}^{\infty} mP_1(i,m,t), \quad i=0,1, \quad n_{2i}(t) = \sum_{m=0}^{\infty} mP_2(i,m,t), \quad i=0,1,2, \tag{12}
$$

and

$$
n_1(t) = n_{10}(t) + n_{11}(t), \qquad n_2(t) = n_{20}(t) + n_{21}(t) + n_{22}(t).
$$

After expressing $P_1(k,t)$ and $P_2(k,t)$ in (10) as the sums of the basic probabilities defined in (6)-(7), we differentiate $\omega_1(t)$ in (10). Then substituting the master equations (1) and (2) gives

$$
\begin{aligned}
\omega_1'(t) =& \sum_{m=0}^{\infty} m^2 P_1'(m,t) = \sum_{m=0}^{\infty} m^2\Big[P_1'(0,m,t) + P_1'(1,m,t)\Big] \\
=& \sum_{m=0}^{\infty} m^2 \Bigg[\underbrace{\nu_1 P_1(1,m-1,t) - \nu_1 P_1(1,m,t)}_{\text{First term}} + \underbrace{(m+1)\delta_1 P_1(m+1,t) - m\delta_1 P_1(m,t)}_{\text{Second term}} \\
& - \underbrace{\kappa_1 P_1(m,t)}_{\text{Third term}} + \underbrace{\kappa_2 \sum_{n=m}^{\infty}\left(\frac{1}{2}\right)^n \binom{n}{m} P_2(n,t)}_{\text{Forth term}}\Bigg].
\end{aligned}
$$

The first two sums can be simplified as

$$
\begin{aligned}
&\nu_1 \sum_{m=0}^{\infty} m^2\Big[P_1(1,m-1,t) - P_1(1,m,t)\Big] = \nu_1 \sum_{m=0}^{\infty}(2m+1)P_1(1,m,t) = 2\nu_1 n_{11}(t) + \nu_1 P_{11}(t), \\
&\delta_1 \sum_{m=0}^{\infty}\Big[m^2(m+1)P_1(m+1,t) - m^3 P_1(m,t)\Big] = \delta_1 \sum_{m=0}^{\infty}(-2m^2+m)P_1(m,t) = \delta_1 n_1(t) - 2\delta_1\omega_1(t).
\end{aligned}
$$

The third sum is simply $\kappa_1\omega_1(t)$, and the last one is

$$
\begin{aligned}
&\kappa_2 \sum_{m=0}^{\infty} m^2 \cdot \sum_{n=m}^{\infty}\left(\frac{1}{2}\right)^n \binom{n}{m} P_2(n,t) = \kappa_2 \sum_{n=0}^{\infty}\left(\frac{1}{2}\right)^n P_2(n,t) \sum_{m=0}^{n} m^2 \binom{n}{m} \\
=&\kappa_2 \sum_{n=0}^{\infty}\left(\frac{1}{2}\right)^n P_2(n,t) \cdot \frac{2^n(n^2+n)}{4} = \frac{\kappa_2}{4}\Big[\omega_2(t) + n_2(t)\Big].
\end{aligned}
$$

By putting these simplifications together, we verify the first equation of (11). The second equation can be verified by a similar procedure.

2 The proof of Theorems

We give mathematical proofs of Theorems 1-4 stated in the main context. For convenience, we restate these theorems before giving their proofs.

2.1 The proof of Theorem 1

Theorem 1 *If the transcription of a gene obeys the model described in Figure 1, then the mean transcription level of the gene in a population of isogenic cells at steady-state is*

$$m^* = m_1^* \cdot \frac{\kappa_2}{\kappa_1+\kappa_2} + m_2^* \cdot \frac{\kappa_1}{\kappa_1+\kappa_2}, \tag{13}$$

a linear combination of the mean levels m_1^* in $\mathbb{S}_1$ stage and m_2^* in $\mathbb{S}_2$ stage, and

$$m_1^* = \frac{2\nu_1\lambda_1(\delta_2+\kappa_2)(\lambda_2+\gamma_2+\kappa_2)+2\nu_2\lambda_2\kappa_1(\lambda_1+\gamma_1+\kappa_1)}{[2(\delta_1+\kappa_1)(\delta_2+\kappa_2)-\kappa_1\kappa_2](\lambda_1+\gamma_1+\kappa_1)(\lambda_2+\gamma_2+\kappa_2)}, \tag{14}$$

$$m_2^* = \frac{2\nu_1\lambda_1\kappa_2(\lambda_2+\gamma_2+\kappa_2)+4\nu_2\lambda_2(\delta_1+\kappa_1)(\lambda_1+\gamma_1+\kappa_1)}{[2(\delta_1+\kappa_1)(\delta_2+\kappa_2)-\kappa_1\kappa_2](\lambda_1+\gamma_1+\kappa_1)(\lambda_2+\gamma_2+\kappa_2)}. \tag{15}$$

Proof By the decomposition

$$m(t) = n_1(t) + n_2(t) = P_1(t)m_1(t) + P_2(t)m_2(t),$$

we get $m^* = P_1^* m_1^* + P_2^* m_2^*$. From the analytical form

$$P_1(t) = \frac{\kappa_2}{\kappa_1+\kappa_2} + \frac{\kappa_1}{\kappa_1+\kappa_2}\, e^{-(\kappa_1+\kappa_2)t}$$

derived in the main context, and $P_2(t) = 1 - P_1(t)$, it follows immediately that

$$P_1^* = \frac{\kappa_2}{\kappa_1+\kappa_2} \quad \text{and} \quad P_2^* = \frac{\kappa_1}{\kappa_1+\kappa_2}. \tag{16}$$

This verifies (13).

It remains to verify (14) and (15). Recall from the main text the definition

$$P_1(t) = P_{10}(t) + P_{11}(t), \quad P_2(t) = P_{20}(t) + P_{21}(t) + P_{22}(t), \tag{17}$$

and the closed system of $P_{1i}(t)$ and $P_{2i}(t)$,

$$\begin{cases} P'_{10}(t) = \kappa_2 P_2(t) + \gamma_1 P_{11}(t) - (\lambda_1+\kappa_1)P_{10}(t), \\ P'_{11}(t) = \lambda_1 P_{10}(t) - (\gamma_1+\kappa_1)P_{11}(t), \\ P'_{20}(t) = \kappa_1 P_1(t) + \gamma_2 P_{21}(t) - (2\lambda_2+\kappa_2)P_{20}(t), \\ P'_{21}(t) = 2\lambda_2 P_{20}(t) - (\lambda_2+\gamma_2+\kappa_2)P_{21}(t) + 2\gamma_2 P_{22}(t), \\ P'_{22}(t) = \lambda_2 P_{21}(t) - (2\gamma_2+\kappa_2)P_{22}(t). \end{cases} \tag{18}$$

From $P_1(t) = P_{10}(t) + P_{11}(t)$ in (17) and the second equation in (18), we find $P_{10}^* + P_{11}^* = P_1^*$, $\lambda_1 P_{10}^* - (\gamma_1+\kappa_1)P_{11}^* = 0$, and therefore

$$P_{11}^* = \frac{\lambda_1\kappa_2}{(\lambda_1+\gamma_1+\kappa_1)(\kappa_1+\kappa_2)}. \tag{19}$$

By taking limit in (17), in the third and the fourth equations in (18), we derive

$$
\begin{aligned}
P_{20}^* + P_{21}^* + P_{22}^* =& P_2^*, \\
(2\lambda_2 + \kappa_2)P_{20}^* - \gamma_2 P_{21}^* =& \kappa_1 P_1^*, \\
2\lambda_2 P_{20}^* - (\lambda_2 + \gamma_2 + \kappa_2)P_{21}^* + 2\gamma_2 P_{22}^* =& 0.
\end{aligned} \tag{20}
$$

As P_1^* and P_2^* are given in (16), we can solve this linear system to obtain P_{20}^*, P_{21}^*, and P_{22}^*, from which it follows that

$$
P_{21}^* + 2P_{22}^* = \frac{2\lambda_2\kappa_1}{(\lambda_2 + \gamma_2 + \kappa_2)(\kappa_1 + \kappa_2)}. \tag{21}
$$

From (9), we find that the steady-states of $n_1(t)$ and $n_2(t)$ satisfy

$$
(\delta_1 + \kappa_1)n_1^* - \frac{\kappa_2}{2}n_2^* = \nu_1 P_{11}^* \quad \text{and} \quad \kappa_1 n_1^* - (\delta_2 + \kappa_2)n_2^* = -\nu_2\left(P_{21}^* + 2P_{22}^*\right). \tag{22}
$$

Thus, n_1^* and n_2^* can be determined by P_{11}^* and $P_{21}^* + 2P_{22}^*$ as

$$
n_1^* = \frac{2\nu_1(\delta_2 + \kappa_2)P_{11}^* + \nu_2\kappa_2(P_{21}^* + 2P_{22}^*)}{2(\delta_1 + \kappa_1)(\delta_2 + \kappa_2) - \kappa_1\kappa_2}, \quad n_2^* = \frac{2\nu_1\kappa_1 P_{11}^* + 2\nu_2(\delta_1 + \kappa_1)(P_{21}^* + 2P_{22}^*)}{2(\delta_1 + \kappa_1)(\delta_2 + \kappa_2) - \kappa_1\kappa_2}. \tag{23}
$$

The final expressions of n_1^* and n_2^* in terms of the system parameters can be obtained by substituting (19) and (21) into (23). The expressions (14) and (15) are then derived from the relations $n_1^* = P_1^* m_1^*$ and $n_2^* = P_2^* m_2^*$. □

2.2 The proof of Theorem 2

Theorem 2 *If the transcription of a gene obeys the model described in Figure 1, then the second moment of its mRNA copy number $M(t)$ at steady-state is*

$$
\mu^* = \mu_1^* \cdot \frac{\kappa_2}{\kappa_1 + \kappa_2} + \mu_2^* \cdot \frac{\kappa_1}{\kappa_1 + \kappa_2}, \tag{24}
$$

where μ_1^* and μ_2^* are the second moments in $\mathbb{S}_1$ and $\mathbb{S}_2$ stages given by

$$
\mu_1^* = m_1^* + \frac{8\nu_1(\kappa_2 + 2\delta_2)\cdot m_{s1}^* + 2\nu_2\kappa_1 \cdot m_{s2}^*}{4(\kappa_1 + 2\delta_1)(\kappa_2 + 2\delta_2) - \kappa_1\kappa_2}, \tag{25}
$$

$$
\mu_2^* = m_2^* + \frac{8\nu_1\kappa_2 \cdot m_{s1}^* + 8\nu_2(\kappa_1 + 2\delta_1)\cdot m_{s2}^*}{4(\kappa_1 + 2\delta_1)(\kappa_2 + 2\delta_2) - \kappa_1\kappa_2}, \tag{26}
$$

with

$$
m_{s1}^* = \frac{(\delta_1 + \lambda_1 + \kappa_1)m_1^* - \kappa_1 m_2^*/2}{\delta_1 + \lambda_1 + \gamma_1 + \kappa_1}, \quad m_{s2}^* = \frac{(\delta_2 + \kappa_2 + 2\lambda_2)m_2^* - \kappa_2 m_1^* + 2\nu_2 p_{22}^*}{\delta_2 + \lambda_2 + \gamma_2 + \kappa_2}, \tag{27}
$$

and $p_{22}^* = 2\lambda_2^2/\left[(\kappa_2 + \lambda_2 + \gamma_2)(\kappa_2 + 2\lambda_2 + 2\gamma_2)\right]$.

Proof In view of P_1^* and P_2^* given in (16), it is clear that (24) follows from the decomposition

$$
\mu(t) = \omega_1(t) + \omega_2(t) = P_1(t)\mu_1(t) + P_2(t)\mu_2(t).
$$

It remains to verify (25) and (26). Recall the following system of $n_{1i}(t)$ and $n_{2i}(t)$:

$$\begin{cases} n'_{10}(t) = \frac{\kappa_2}{2} n_2(t) - (\delta_1 + \lambda_1 + \kappa_1) n_{10}(t) + \gamma_1 n_{11}(t), \\ n'_{11}(t) = \lambda_1 n_{10}(t) + \nu_1 P_{11}(t) - (\delta_1 + \gamma_1 + \kappa_1) n_{11}(t), \\ n'_{20}(t) = \kappa_1 n_1(t) + \gamma_2 n_{21}(t) - (\delta_2 + 2\lambda_2 + \kappa_2) n_{20}(t), \\ n'_{21}(t) = 2\lambda_2 n_{20}(t) + 2\gamma_2 n_{22}(t) + \nu_2 P_{21}(t) - (\delta_2 + \lambda_2 + \gamma_2 + \kappa_2) n_{21}(t), \\ n'_{22}(t) = \lambda_2 n_{21}(t) + 2\nu_2 P_{22}(t) - (\delta_2 + 2\gamma_2 + \kappa_2) n_{22}(t), \end{cases} \tag{28}$$

From the definition of $n_1(t)$ in (8) and the first equation in (28), we find

$$n^*_{10} + n^*_{11} = n^*_1, \quad (\delta_1 + \lambda_1 + \kappa_1) n^*_{10} - \gamma_1 n^*_{11} = \frac{\kappa_2}{2} n^*_2,$$

and therefore

$$n^*_{11} = \frac{2(\delta_1 + \lambda_1 + \kappa_1) n^*_1 - \kappa_2 n^*_2}{2(\delta_1 + \lambda_1 + \gamma_1 + \kappa_1)}. \tag{29}$$

By taking limit in (8), the third and the last equations in (28), we derive

$$\begin{aligned} n^*_{20} + n^*_{21} + n^*_{22} =& n^*_2, \\ (\delta_2 + 2\lambda_2 + \kappa_2) n^*_{20} - \gamma_2 n^*_{21} =& \kappa_1 n^*_1, \\ \lambda_2 n^*_{21} - (\delta_2 + 2\gamma_2 + \kappa_2) n^*_{22} =& -2\nu_2 P^*_{22}. \end{aligned}$$

We can solve this linear system to express n^*_{20}, n^*_{21} and n^*_{22} as functions of n^*_1, n^*_2, and P^*_{22}, from which it follows that

$$n^*_{21} + 2n^*_{22} = \frac{(\delta_2 + 2\lambda_2 + \kappa_2) n^*_2 - \kappa_1 n^*_1 + 2\nu_2 P^*_{22}}{\delta_2 + \lambda_2 + \gamma_2 + \kappa_2}, \tag{30}$$

where n^*_1 and n^*_2 are given explicitly in (23), and by solving the linear system (20),

$$P^*_{22} = \frac{2\lambda_2^2 \kappa_1}{(\kappa_2 + \lambda_2 + \gamma_2)(\kappa_2 + 2\lambda_2 + 2\gamma_2)(\kappa_1 + \kappa_2)}. \tag{31}$$

From (11), we find that the steady-states of $\omega_1(t)$ and $\omega_2(t)$ satisfy

$$\begin{aligned} (2\delta_1 + \kappa_1)\omega^*_1 - \frac{\kappa_2}{4}\omega^*_2 =& \delta_1 n^*_1 + \frac{\kappa_2}{4} n^*_2 + \nu_1 \Big[2n^*_{11} + P^*_{11} \Big], \\ -\kappa_1 \omega^*_1 + (2\delta_2 + \kappa_2)\omega^*_2 =& \delta_2 n^*_2 + \nu_2 \Big[P^*_{21} + 2P^*_{22} + 2n^*_{21} + 4n^*_{22} \Big]. \end{aligned}$$

Thus, ω^*_1 and ω^*_2 can be expressed as functions of n^*_1, n^*_2, P^*_{11}, $P^*_{21} + 2P^*_{22}$, n^*_{11}, and $n^*_{21} + 2n^*_{22}$. By using (22), we can express P^*_{11} and $P^*_{21} + 2P^*_{22}$ as linear combinations of n^*_1 and n^*_2, and

$$\omega^*_1 = n^*_1 + \frac{8\nu_1(\kappa_2 + 2\delta_2) n^*_{11} + 2\nu_2 \kappa_2 (n^*_{21} + 2n^*_{22})}{4(\kappa_1 + 2\delta_1)(\kappa_2 + 2\delta_2) - \kappa_1 \kappa_2}, \tag{32}$$

$$\omega^*_2 = n^*_2 + \frac{8\nu_1 \kappa_1 n^*_{11} + 8\nu_2 (\kappa_1 + 2\delta_1)(n^*_{21} + 2n^*_{22})}{4(\kappa_1 + 2\delta_1)(\kappa_2 + 2\delta_2) - \kappa_1 \kappa_2}. \tag{33}$$

By introducing

$$m^*_{s1} = \frac{n^*_{11}}{P^*_1} \quad \text{and} \quad m^*_{s2} = \frac{n^*_{21} + 2n^*_{22}}{P^*_2}, \tag{34}$$

and dividing (32) and (33) by P_1^* and P_2^* respectively, we derive

$$\mu_1^* = \frac{\omega_1^*}{P_1^*} = m_1^* + \frac{8\nu_1(\kappa_2+2\delta_2)m_{s1}^* + 2\nu_2 m_{s2}^* \cdot \kappa_2 P_2^*/P_1^*}{4(\kappa_1+2\delta_1)(\kappa_2+2\delta_2)-\kappa_1\kappa_2},$$
$$\mu_2^* = \frac{\omega_2^*}{P_2^*} = m_2^* + \frac{8\nu_1 m_{s1}^* \cdot \kappa_1 P_1^*/P_2^* + 8\nu_2(\kappa_1+2\delta_1)m_{s2}^*}{4(\kappa_1+2\delta_1)(\kappa_2+2\delta_2)-\kappa_1\kappa_2},$$

from which (25) and (26) follow immediately because (16) implies

$$\kappa_2 P_2^*/P_1^* = \kappa_1 \qquad \text{and} \qquad \kappa_1 P_1^*/P_2^* = \kappa_2.$$

From (29) we find

$$\begin{aligned} m_{s1}^* =& \frac{2(\delta_1+\lambda_1+\kappa_1)n_1^*/P_1^* - \kappa_2 n_2^*/P_1^*}{2(\delta_1+\lambda_1+\gamma_1+\kappa_1)} \\ =& \frac{2(\delta_1+\lambda_1+\kappa_1)m_1^* - \kappa_2 P_2^*/P_1^* \cdot m_2^*}{2(\delta_1+\lambda_1+\gamma_1+\kappa_1)} \\ =& \frac{2(\delta_1+\lambda_1+\kappa_1)m_1^* - \kappa_1 m_2^*}{2(\delta_1+\lambda_1+\gamma_1+\kappa_1)}, \end{aligned}$$

and verify the first part in (27). From (30) we have

$$\begin{aligned} m_{s2}^* =& \frac{(\delta_2+2\lambda_2+\kappa_2)n_2^*/P_2^* - \kappa_1 n_1^*/P_2^* + 2\nu_2 P_{22}^*/P_2^*}{\delta_2+\lambda_2+\gamma_2+\kappa_2} \\ =& \frac{(\delta_2+2\lambda_2+\kappa_2)m_2^* - \kappa_2 m_1^* + 2\nu_2 p_{22}^*}{\delta_2+\lambda_2+\gamma_2+\kappa_2}, \end{aligned}$$

and verify the second part in (27). The expression of $p_{22}^* = P_{22}^*/P_2^*$ is derived from (16) and (31). □

2.3 The proof of Theorem 3

By (14) and (15), the ratio of mRNA copy number at steady-state in $\mathbb{S}_2$ stage to that in $\mathbb{S}_1$ stage is given by

$$r^* = \frac{m_2^*}{m_1^*} = \frac{\nu_1\lambda_1\kappa_2(\lambda_2+\gamma_2+\kappa_2) + 2\nu_2\lambda_2(\delta_1+\kappa_1)(\lambda_1+\gamma_1+\kappa_1)}{\nu_1\lambda_1(\delta_2+\kappa_2)(\lambda_2+\gamma_2+\kappa_2) + \nu_2\lambda_2\kappa_1(\lambda_1+\gamma_1+\kappa_1)}. \tag{35}$$

In order to emphasize the impact of the cell cycle stage transition on the variation of r^*, we consider the case that the transcription kinetics are unchanged in the two stages:

$$\nu_i = \nu,\ \delta_i = \delta,\ \lambda_i = \lambda,\ \gamma_i = \gamma,\quad i = 1, 2. \tag{36}$$

When it holds, we can simplify (35) to the form

$$r^* = \frac{2(\delta+\kappa_1)(\lambda+\gamma+\kappa_1) + \kappa_2(\lambda+\gamma+\kappa_2)}{\kappa_1(\lambda+\gamma+\kappa_1) + (\delta+\kappa_2)(\lambda+\gamma+\kappa_2)}. \tag{37}$$

It is interesting to see that the fold change r^* in (37) depends on $\lambda+\gamma$, but not on λ and γ individually, and is independent of the synthesis rate ν.

Theorem 3 *For any constant $C > 0$, there exist system parameters under the constraint (36) to make $r^* = C$.*

Proof We prove the result by specifying the parameter sets to make $r^* = C$ for C in different ranges. First, we consider the case that $C \geq 2$, and choose

$$\kappa_1 = C\kappa_2, \quad \lambda + \gamma = \kappa_2, \quad \delta = \frac{(C^3 - C^2 - 2)\kappa_2}{2}.$$

Note that $\delta > 0$ since $C \geq 2$ implies $C^3 - C^2 - 2 \geq 2C^2 - C^2 - 2 \geq C^2 - 2 > 0$. It follows from (37) that

$$\begin{aligned} r^* =& \frac{2\big[(C^3 - C^2 - 2)\kappa_2/2 + C\kappa_2)\big](C\kappa_2 + \kappa_2) + 2\kappa_2^2}{C\kappa_2(C\kappa_2 + \kappa_2) + (2\kappa_2(C^3 - C^2 - 2)\kappa_2/2)} \\ =& \frac{\big[(C^3 - C^2 - 2) + 2C\big](C+1)\kappa_2^2 + 2\kappa_2^2}{C(C+1)\kappa_2^2 + (C^3 - C^2 - 2)\kappa_2^2} = C. \end{aligned}$$

When $C \leq 1$, we take

$$\lambda + \gamma = \kappa_1, \quad \kappa_2 = \frac{4\kappa_1}{C}, \quad \delta = \frac{(16 - 12C - 2C^3)\kappa_1}{C^3} > 0.$$

Substituting these parameters into (37), we have

$$\begin{aligned} r^* =& \frac{2[(16 - 12C - 2C^3)\kappa_1/C^3 + \kappa_1](\kappa_1 + \kappa_1) + 4\kappa_1 C \cdot (\kappa_1 + 4\kappa_1/C)}{\kappa_1(\kappa_1 + \kappa_1) + [(16 - 12C - 2C^3)\kappa_1/C^3 + 4\kappa_2/C](\kappa_1 + 4\kappa_1/C)} \\ =& \frac{4(16 - 12C - C^3)\kappa_1^2/C^3 + 4(C+4)\kappa_1^2/C^2}{2\kappa_1^2 + (C+4)(16 - 12C + 4C^2 - 2C^3)\kappa_1^2/C^4} = C. \end{aligned}$$

When $C \in (3/2, 2)$, we choose

$$\lambda + \gamma = \kappa_1 = \kappa_2, \quad \delta = \frac{(2C - 3)\kappa_2}{2 - C} > 0.$$

By (37), we derive

$$r^* = \frac{2[(2C - 3)\kappa_2/(2 - C) + \kappa_2] \cdot 2\kappa_2 + 2\kappa_2^2}{2\kappa_2^2 + [(2C - 3)\kappa_2/(2 - C) + \kappa_2] \cdot 2\kappa_2} = C.$$

Finally, we consider the case that $C \in (1, 3/2]$, and choose

$$\lambda + \gamma = 2\kappa_2, \quad \kappa_1 = (C - 1)\kappa_2, \quad \delta = \frac{(C - 1)(C^2 - C + 1)\kappa_2}{2 - C} > 0.$$

By (37), we derive

$$r^* = \frac{2[(C - 1)(C^2 - C + 1)\kappa_2/(2 - C) + (C - 1)\kappa_2] \cdot (C + 1)\kappa_2 + 3\kappa_2^2}{(C - 1)\kappa_2 \cdot (C + 1)\kappa_2 + [(C - 1)(C^2 - C + 1)\kappa_2/(2 - C) + \kappa_2] \cdot 3\kappa_2} = C. \quad \square$$

2.4 The proof of Theorem 4

Theorem 4 *Let (36) hold. Then we have*

(a) When κ_1 increases from 0 to ∞, r^* increases from $r^*(0,\kappa_2) < 2$ until it peaks uniquely and then decreases to approach 2 at ∞. In particular, $r^* > 2$ if and only if

$$\kappa_1 > \kappa_2 + \frac{\kappa_2(\kappa_2 + \lambda + \gamma)}{2\delta}. \tag{38}$$

(b) When κ_2 increases from 0 to ∞, r^* decreases from $r^*(\kappa_1, 0) > 2$ until it bottoms out uniquely and then increases to approach 1 at ∞. In particular, $r^* < 1$ if and only if

$$\kappa_2 > 2\kappa_1 + \lambda + \gamma + \frac{\kappa_1(\lambda + \gamma + \kappa_1)}{\delta}. \tag{39}$$

(c) When $\kappa_1 \le \kappa_2$, r^* has an upper bound strictly less than 2.

Proof (a). From (37) we find

$$\begin{aligned} r^*(0,\kappa_2) =& \frac{2\delta(\lambda+\gamma) + \kappa_2(\lambda+\gamma+\kappa_2)}{(\delta+\kappa_2)(\lambda+\gamma+\kappa_2)} \\ =& \frac{2(\delta+\kappa_2)(\lambda+\gamma+\kappa_2) - \kappa_2(2\delta+\lambda+\gamma+\kappa_2)}{(\delta+\kappa_2)(\lambda+\gamma+\kappa_2)} \\ =& 2 - \frac{\kappa_2(2\delta+\lambda+\gamma+\kappa_2)}{(\delta+\kappa_2)(\lambda+\gamma+\kappa_2)} < 2. \end{aligned}$$

Differentiating (37) with respect to κ_1 gives

$$\frac{\partial r^*(\kappa_1,\kappa_2)}{\partial \kappa_1} = \frac{-2\delta(\lambda+\gamma+\kappa_1)^2 + (\lambda+\gamma+\kappa_2)[2\delta(\lambda+\gamma+2\kappa_1+\kappa_2+\delta) + \kappa_2(\lambda+\gamma+2\kappa_1)]}{[\kappa_1(\lambda+\gamma+\kappa_1) + (\delta+\kappa_2)(\lambda+\gamma+\kappa_2)]^2}.$$

For convenience, we write its numerator as $h(\kappa_1)$ for a moment. Then

$$h(0) = \kappa_2(\lambda+\gamma)(2\delta+\lambda+\gamma+\kappa_2) + 2\delta(\delta+\kappa_2)(\lambda+\gamma+\kappa_2) > 0,$$

indicating that r^* increases for small $\kappa_1 > 0$. Since $h(\kappa_1)$ is a quadratic function of κ_1 with the leading coefficient $-2\delta < 0$ and $h(0) > 0$, it vanishes exactly once in $(0,\infty)$, at which r^* peaks uniquely, and after which r^* decreases and tends to its limit 2 at ∞.

To verify the last part, we note that $r^* > 2$ if and only if

$$\begin{aligned} & 2(\delta+\kappa_1)(\lambda+\gamma+\kappa_1) + \kappa_2(\lambda+\gamma+\kappa_2) - 2\kappa_1(\lambda+\gamma+\kappa_1) - 2(\delta+\kappa_2)(\lambda+\gamma+\kappa_2) \\ &= 2\delta(\lambda+\gamma+\kappa_1) - (2\delta+\kappa_2)(\lambda+\gamma+\kappa_2) \\ &= [2\delta\kappa_1 - 2\delta\kappa_2 - \kappa_2(\lambda+\gamma+\kappa_2)] > 0. \end{aligned}$$

Clearly, it is equivalent to (38).

(b). By using a similar argument as in the proof of (a), we can show that $r^*(\kappa_1, 0) > 2$. Differentiating (37) with respect to κ_2 gives

$$\frac{\partial r^*(\kappa_1,\kappa_2)}{\partial \kappa_2} = \frac{\delta(\lambda+\gamma+\kappa_2)^2 - (2\delta+\kappa_1)(\lambda+\gamma+2\kappa_2)(\lambda+\gamma+\kappa_1) - 2\delta(\delta+\kappa_1)(\lambda+\gamma+\kappa_1)}{[\kappa_1(\lambda+\gamma+\kappa_1) + (\delta+\kappa_2)(\lambda+\gamma+\kappa_2)]^2}.$$

Let $g(\kappa_2)$ denote its numerator for a moment. Then

$$g(0) = -(\delta+\kappa_1)(\lambda+\gamma)^2 - \kappa_1(\lambda+\gamma)(2\delta+\kappa_1) - 2\delta(\delta+\kappa_1)(\lambda+\gamma+\kappa_1) < 0,$$

indicating that r^* decreases for $\kappa_2 > 0$ small. Since the quadratic function $g(\kappa_2)$ has the leading coefficient $\delta > 0$ and $g(0) < 0$, it vanishes exactly once in $(0, +\infty)$, at which r^* bottoms out uniquely, and after which r^* increases and tends to its limit 1 at infinity. Finally, $r^* < 1$ is equivalent to

$$\begin{aligned}
&2(\delta+\kappa_1)(\lambda+\gamma+\kappa_1) + \kappa_2(\lambda+\gamma+\kappa_2) - \kappa_1(\lambda+\gamma+\kappa_1) - (\delta+\kappa_2)(\lambda+\gamma+\kappa_2)\\
&= (2\delta+\kappa_1)(\lambda+\gamma+\kappa_1) - \delta(\lambda+\gamma+\kappa_2)\\
&= \delta(\lambda+\gamma) + \kappa_1(2\delta+\lambda+\gamma+\kappa_1) - \delta\kappa_2 < 0.
\end{aligned}$$

(c). From the proof of (a),

$$\begin{aligned}
h(\kappa_1) =& -2\delta(\lambda+\gamma+\kappa_1)^2 + (\lambda+\gamma+\kappa_2)[2\delta(\lambda+\gamma+2\kappa_1+\kappa_2+\delta) + \kappa_2(\lambda+\gamma+2\kappa_1)]\\
\geq & -2\delta(\lambda+\gamma+\kappa_1)^2 + (\lambda+\gamma+\kappa_2)\cdot 2\delta(\lambda+\gamma+2\kappa_l+\kappa_2+\delta)\\
=& \, 2\delta(\kappa_2-\kappa_1)(\lambda+\gamma+\kappa_1) + 2\delta(\lambda+\gamma+\kappa_2)(\kappa_1+\kappa_2+\delta) > 0
\end{aligned}$$

holds for all $\kappa_1 \leq \kappa_2$. Thus r^* increases in $(0, \kappa_2)$, indicating that

$$r^*(\kappa_1, \kappa_2) \leq r^*(\kappa_2, \kappa_2) = \frac{2\delta+3\kappa_2}{\delta+2\kappa_2} < 2.$$

The proof is completed. $\square$
